# Combating White Spot Syndrome Virus (WSSV) in Global Shrimp Farming: Unraveling Its Biology, Pathology, and Control Strategies

**DOI:** 10.3390/v17111463

**Published:** 2025-10-31

**Authors:** Md. Iftehimul, Neaz A. Hasan, David Bass, Abul Bashar, Mohammad Mahfujul Haque, Morena Santi

**Affiliations:** 1 Department of Biotechnology, Bangladesh Agricultural University, Mymensingh 2202, Bangladesh; iftehimul.23221212@bau.edu.bd; 2 Department of Fisheries and Marine Bioscience, Gopalganj Science and Technology University, Gopalganj 8100, Bangladesh; 3 Centre for Environment, Fisheries and Aquaculture Science (Cefas), Barrack Road, The Nothe Weymouth, Dorset DT4 8UB, UK; 4 Department of Aquaculture, Bangladesh Agricultural University, Mymensingh 2202, Bangladesh

**Keywords:** WSSV, shrimp farming, co-infection, transmission dynamics, immunology

## Abstract

White Spot Syndrome Virus (WSSV) is one of the most devastating viral pathogens affecting shrimp, causing severe economic losses to the global farmed shrimp trade. The globalization of live shrimp trade and waterborne transmission have facilitated the rapid spread of WSSV across major shrimp-producing countries since its initial emergence. The present review gives an updated account of WSSV biology, pathology, transmission dynamics, and recent developments in control measures. The virus, a double-stranded DNA virus of the *Nimaviridae* family, utilizes advanced immune evasion strategies, resulting in severe mortality. Shrimp lack adaptive immunity and hence rely predominantly on innate immunity, which is insufficient to mount an effective response against severe infections. Traditional disease control measures such as augmented biosecurity, selective breeding, and immunostimulants have, despite extensive research, achieved only limited success. New biotechnological tools such as RNA interference, CRISPR-Cas gene editing, and nanotechnology offer tremendous potential for disease mitigation. In parallel, the development of DNA and RNA vaccines targeting WSSV structural proteins, such as VP28, holds significant promise for stimulating the shrimp immune system. This review highlights the urgent need for a convergent approach to sustainable disease management in global shrimp aquaculture, with interdisciplinarity playing a pivotal role in shaping the future of WSSV control.

## 1. Introduction

**Figure 1:**
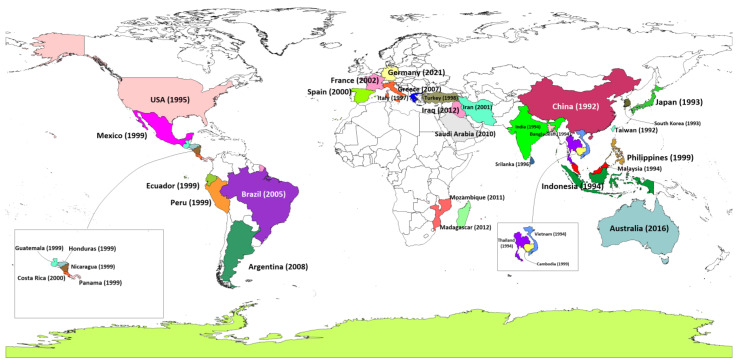
Global distribution of White Spot Syndrome Virus (WSSV) disease.

Shrimp aquaculture emerged as an international enterprise during the latter part of the 20th century, delivering nutrition- and protein-dense seafood to meet growing global market demand. With the boom of shrimp farming throughout much of Asia and beyond came a formidable foe: White Spot Syndrome Virus (WSSV), now one of the most virulent pathogens of shrimp in aquaculture. Since the emergence of WSSV in 1992, the global shrimp sector has suffered an estimated USD 8–15 billion in economic losses from this single disease [1,2]. For the Asian shrimp industry, a loss of about USD 20 billion due to WSSV was possibly its worst experience [3]. The widespread presence of WSSV in global shrimp farms is a continuing nightmare for farmers. The virus can persist in pond sediments and surrounding areas for over twenty months, with studies detecting its presence in ponds soil for over ten months post-outbreak [4]. Notably, water serves as a critical medium for rapid viral dissemination; research has shown that WSSV DNA can be detected in water within six hours of disease onset in shrimp, with shedding intensifying until the host’s death [5]. Early reports of this disease from shrimp farms in China, Thailand, Vietnam, Indonesia, Ecuador, and many countries that are among the largest producers of farmed shrimp in the world (Figure 1). The speed with which the virus spread also reflected how quickly, and across borders, live shrimp and possibly contaminated water were traded in the globalized shrimp industry. By the early 2000s, WSSV had spread without control in shrimp aquaculture and epidemics appeared to be underway in almost all major shrimp-producing countries globally [6]. While the devastating impacts of the virus were felt worldwide by shrimp farmers and aquaculture scientists, the virus continued to evolve, infecting new species and gradually adapting to different environmental conditions. Over the years, WSSV advanced so rapidly from farms in Bangladesh to shrimp ponds in Brazil that the industry called for more sustainable farming practices paired with effective disease management [7].

The biology of WSSV provides insight into how efficiently it wreaks havoc as a pathogen. It is a double-stranded DNA virus from the *Nimaviridae* family, and expresses a large number of proteins that facilitate immune evasion, manipulation of host physiology, and rapid replication characteristics [8]. The ability of the virus to infect a range of crustaceans including shrimp, crabs, and even non-crustacean carriers [9], highlights its adaptability and cosmopolitanism. The structural proteins of WSSV, especially the envelope proteins VP28 and VP26, are critical in the infection process by interacting with receptors on host cells to facilitate entry [10]. Once the virus enters, it exploits the host cell machinery to replicate itself and quickly spread through shrimp tissues, leading to systemic infection [1]. Due to the absence of adaptive immunity in shrimp, this pathogen readily overwhelms the immune physiology of its crustacean host [11]. Infected organisms quickly die as the virus multiplies, and the disease spreads through aquaculture systems. One of the biggest hindrances in controlling outbreaks of WSSV are their complex transmission dynamics. Moreover, the virus can remain viable over a wide range of salinities and temperatures, which makes control more difficult, especially in areas of environmental fluctuations [12]. WSSV causes USD 19 billion in losses, annually, across small- and commercial-scale shrimp farms worldwide [12]. For countries such as Thailand, India, or China, which depend heavily on shrimp trading for their revenues, the economic impacts from crop failures have been severe and, in many cases, resulted in bankruptcy. WSSV disease outbreaks also caused global shifts in the shrimp supply chain; nations often stopped domestic shrimp exports for a period of time and/or increased their imports to maintain supplies to local markets [13,14]. WSSV also imposes an environmental cost as some farmers despite the ineffectiveness of antibiotics against viruses use large quantities of antibiotics and other chemicals in bids to control outbreaks. This inappropriate use leads to the accumulation of antibiotic residues in the environment and the rise of antimicrobial resistance [15]. This emphasizes the need for the rapid development of environmentally sustainable strategies for controlling WSSV in aquaculture. Scientists and industry have tested various approaches over several decades to control WSSV, but a single solution has not yet been found. Conventional efforts like selective breeding of disease-resistant shrimp and better management practices have had limited success [16]. Nevertheless, additional development of immunostimulants, probiotics, and essential oils has shown potential in stimulating the natural immunity of shrimp [17]. Some immunostimulants from algae, plants, and even fungi also enhance the innate immune response of shrimp, thus reducing viral infection [18]. Beneficial bacteria that help overall gut health such as probiotics have also entered into assessments for use within shrimp diets as a means to decrease the infectivity of WSSV [19]. These methods are signs of an evolution to more natural and sustainable measures that improve shrimp resistance to diseases, as opposed to the application of chemical treatments. Recent advances in some innovative technologies, such as DNA and RNA vaccines, and nanotechnology offer further tools for effective WSSV mitigation [20]. DNA vaccines utilize specific genes from the virus to provoke an immune response in the animal; targeting critical viral proteins (e.g., VP28) has been shown to be effective in vitro [21]. Moreover, RNA interference (RNAi) technology has been used to target specific WSSV genes, enabling gene silencing and significantly inhibiting viral replication in the shrimp [22]. The specificity and controllability of nanotechnology may open new avenues for the precise delivery of drugs for targeted therapies against WSSV [23], thus improving treatment efficiency and reducing instability under variable environmental conditions. These advancements showcase the promise of biotechnology applications as tools for a sustainable, effective disease management plan in shrimp aquaculture, paving the way to a more robust shrimp supply chain. Considering both the severity of WSSV selective pressure and the multitude of mechanisms involved in its proliferation and circulation, we posit that a multifaceted strategy is required to reach durable control over this pathogen in shrimp aquaculture. Taking a broad perspective from the biology and transmission of WSSV to new control strategies, this review is intended as a one-stop-shop for researchers, farmers, and policymakers struggling to keep pace with the diverse aspects of WSSV research. Drawing on material across disciplines, this review highlights the opportunity for concerted action to produce resilient and sustainable options that will provide solutions capable of future-proofing shrimp aquaculture. With the growing aquaculture industry and demand for shrimp, there is an urgent need to solve the threat posed by WSSV.

## 2. Integrated Review and Analytical Methods

**Figure 2:**
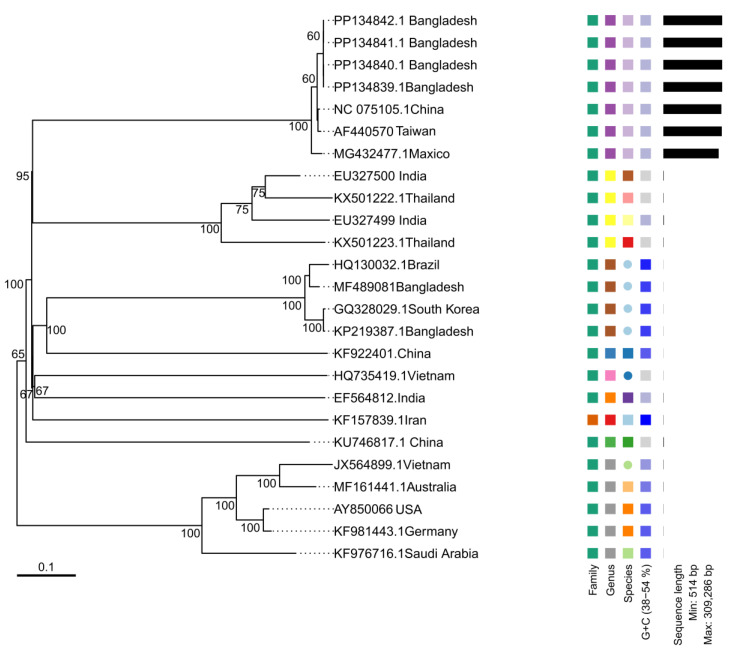
Geographical genetic diversity of WSSV across major affected countries.

This review presents a systematic effort in understanding the biology, pathology, and diagnostic methods as well as control measures associated with White Spot Syndrome Virus (WSSV), a globally important pathogen of shrimp aquaculture. Data were synthesized according to a structured methodology adopted from various primary and secondary sources. A semi-structured search of the relevant literature was conducted using the databases of Web of Science, Scopus, PubMed, and Google Scholar, focusing on peer-reviewed articles, government reports, and industry publications. Keywords used in the database search included variations and combinations of “WSSV diagnostics”, “shrimp viral diseases”, “aquaculture sustainability”, and “viral transmission in crustaceans”. After reviewing these primary sources, additional literature was identified by examining references cited in the initial studies, as well as through subsequent non-systematic searches on Google Scholar, Web of Science, and ScienceDirect. The Preferred Reporting Items for Systematic Reviews and Meta-Analyses (PRISMA) guidelines were used to further transparency in the entire selection process. A total of 202 articles were found, and the final number of articles that were considered relevant for analysis was 108 after removal of duplicates and articles not relevant. These articles gave insights on the historical emergence and global distribution of WSSV and the biological mechanisms of its infection. To enhance the scope of the review, spatial and phylogenetic analyses were performed using secondary data. GPS coordinates of WSSV-infected zones were collected from government and non-government databases and visualized using ESRI’s ArcGIS software (version 10.8). A phylogenetic tree (Figure 2) was constructed from the whole-genome sequences of WSSV isolates retrieved from NCBI GenBank, analyzed using the VICTOR platform [24]. This infers taxonomy ranks (family, genus, species) from genome-based distance values according to ICTV standards; in this case, all isolates belong to the same viral species, and the rank labels are formal classification markers and not for divergent groups. Moreover, the review covers the economic and ecological impact assessments of WSSV outbreaks and these were conducted by compiling the global production statistics and analyzing co-infection reports with other pathogens. Such an integrative approach allowed the identification of patterns in WSSV spread and resilience mechanisms in shrimp.

## 3. History of White Spot Syndrome Virus

Shrimp is one of the most valuable species in global aquaculture, prized for its high levels of protein, omega-3 fatty acids, vitamins, and minerals [25]. The development of modern shrimp farming began in Japan, where Fujinaga pioneered semi-intensive shrimp farming techniques in the mid-20th century [26]. His innovations, including advancements in shrimp spawning, larval rearing, and growth techniques, laid the groundwork for the expansion of shrimp farming to other regions such as Taiwan, regions of China, and the United States [27]. As semi-intensive shrimp farming techniques were adopted globally, inputs such as feed, therapeutic agents, and overstocking were introduced without proper regulation, resulting in outbreaks of various diseases (e.g., White Spot Syndrome Virus (WSSV), *Enterocytozoon hepatopenaei* (EHP), infectious hypodermal and hematopoietic necrosis (IHHNV), infectious myonecrosis virus (IMNV), yellow head virus (YHV), Taura syndrome virus (TSV), *Macrobrachium rosenbergii nodavirus* (MrNV), and acute hepatopancreatic necrosis disease (AHPND)) [28,29]. Among these, WSSV has caused the most devastating financial losses with mortality rates up to 100% within 7–10 days of infection [30]. WSSV was first reported in 1992 in cultured *Penaeus japonicas* in Taiwan and China [31,32], quickly spreading to Japan and Korea by 1993 where the disease was reported in farmed *Peaeaus japonicas* and *Penaeus orientalis*, respectively [33,34]. The rapidity in which WSSV spread across Asia (Table 1) caused massive devastation to the shrimp aquaculture industry, conservatively estimated at billions of dollars in losses, severely affecting the local economies of those countries. This single disease caused annual losses of over USD 500 million in China during its first prevalence due to reduced shrimp yields [35]. This outbreak seriously affected the global supply of shrimp while China was one of the large shrimp producers at the time and continued to maintain its production pace. By 1994, the virus spread throughout Southeast Asia, affecting countries such as Thailand, Malaysia, Indonesia, Vietnam, India, and Bangladesh [26,36,37,38]. During this period, Thailand was the world’s largest shrimp producer, and it was estimated that outbreak caused losses of approximately USD 600 million within one year, crippling the aquaculture industry [39]. In India, the virus caused annual economic losses exceeding USD 100 million due to a more than 80% reduction in shrimp exports [40]. The spread of virus in Vietnam resulted in annual losses of approximately USD 200 million, seriously damaging the nation’s economy [39]. For Indonesia, which ranked second globally as a shrimp-producing country by 2001, WSSV induced annual losses ranging between USD 300 million and USD 400 million [41]. In Bangladesh, the first major outbreak occurred in semi-intensive shrimp farms in Cox’s Bazar in 1994, primarily affecting *Penaeus monodon*. The outbreak led to widespread devastation, with 90% of shrimp farms impacted, resulting in a 20% decrease in national shrimp production. A subsequent outbreak in 2001, driven by unplanned and uncontrolled expansion of shrimp farming, affected 25% of production [42]. Since 2007, the frequency of outbreaks in Bangladesh has increased, with WSSV remaining the leading cause of production loss [7]. The spread of WSSV was not confined to Asia. By 1995, the virus had reached the United States, likely introduced through frozen shrimp imports. The virus was detected in cultured shrimp in Texas and South Carolina in 1997 and 1998, respectively [43]. Subsequently, in 1999, major WSSV epizootics occurred in Ecuador, Panama, Honduras, Guatemala, Mexico, Cambodia, Nicaragua and South Asian country the Philippines, primarily affecting cultured *P. monodon* [44,45,46,47]. In Ecuador, a major shrimp exporting country in Latin America, losses were estimated at over USD 300 million annually during the early 2000s [48]. WSSV infections were causing losses of more than USD 300 million annually in Mexico during the early years of the outbreak, which prompted widespread adoption of biosecurity measures to reduce the impact of the virus [49].

By 2000, the virus had spread to Costa Rica, where it was first detected in *Litopenaeus vannamei* farms in the Gulf of Nicoya [52]. In the same period (between 1995 and 2001), the virus was detected in shrimp farms in several European Union (EU) countries including Greece, Italy, and Spain, and later in Turkey [53]. In 2002, France reported its first WSSV outbreak, traced back to wild crustaceans [53,54]. The Middle East was affected by WSSV, with the first outbreak in *L. vannamei* reported in the Khuzestan province of Iran in 2001 [55]. Brazil recorded its first WSSV outbreak in *L. vannamei* farms in the Laguna province in 2005 [56], and by 2008, the virus was detected in Argentina [57]. WSSV was reported in Saudi Arabia in 2010 and off the coast of Iraq in wild penaeids in 2012 [58,59]. In Africa, the first detection of WSSV occurred at the Aquapesca shrimp farm in Quelimane, Mozambique, in 2011, with a subsequent outbreak in Madagascar in 2012 [45]. Most recently, in November 2016, WSSV was identified in a prawn farm near Brisbane, Queensland, Australia [60].

## 4. Biology of WSSV

### 4.1. Taxonomy, Evolution and Protein Homology of WSSV with Other Taxa

WSSV was officially named in 2005 after multiple reclassifications [61]. It was described earlier under various names in the literature, including hypodermal and hematopoietic necrosis baculovirus (HHNBV) [62], rod-shaped nuclear virus of *P. japonicus* (RV-PJ) [62], Chinese baculovirus (CBV) [63], systemic ectodermal and mesodermal baculovirus (SEMBV) [64], penaeid rod-shaped DNA virus (PRDV) [65], and white spot baculovirus (WSBV) [62]. Initially, what is now WSSV was considered a non-occluded Baculovirus due to its cylindrical morphological characteristics and histological injuries observed at the onset of the virus [66]. However, it was found to differ genetically and ultrastructurally from them. It was later reclassified as the only member of the genus *Whispovirus*, in the family *Nimaviridae*, by the International Committee of Taxonomy of Viruses on the basis of its thread-like polar extension—the distinguishing morphological feature of the family [8].

WSSV taxonomy thus reflects not only its unique structure but also represents a distant phylogenetic relation to other large dsDNA viruses, which include members of the *Baculoviridae*, *Ascoviridae*, *Asfarviridae*, *phycodnaviridae*, and *Iridoviridae* families [8]. WSSV presents a unique genomic organization and shares a relatively small subset of conserved genes with the earlier aforementioned viral families, indicating a distant evolutionary relationship. Thus, large dsDNA viruses are characterized by comparative phylogenetic studies with genetic conservatism, particularly in genes involved in DNA replication and repair. These observations suggest that WSSV and other virus families may have diverged from other virus families and evolved over time into distinct genomic features. Evidence of this evolutionary linkage is further supported through detailed protein homology analysis, which reflects notable sequence alignments between WSSV proteins and those of other dsDNA viruses. In terms of homologous relationships, wsv459 protein of WSSV shares full identity with a hypothetical protein from PBCV-1 (*Phycodnaviridae*), with an E-value of 3 × 10^−4^. This strong conservation among those viral families suggests that this protein may have a very important role in the virus life cycle. On the other hand, wsv360 and wsv143 are homologous to proteins of *Asfarviridae* and *Ascoviridae*, showing identity values of 86% and 96%, with E-values of 0.008 and 0.022, respectively. The low values of the E-parameter indicate that the observed homologies are statistically significant and are not due to chance alignments of sequences. Probably the most significant information deduced from this is that conserved proteins, such as ribonucleotide reductase, exist across viral families, indicating shared molecular mechanisms crucial for viral replication [67]. Apart from ribonucleotide reductase, other WSSV proteins homologous to those from various viral families like *Poxviridae*, *Mimiviridae*, and *Baculoviridae* are notable. For instance, wsv486 shares 90% identity with the variola B22R protein from FWPV (*Poxviridae*) with an E-value of 0.041, suggesting functional relatedness between these proteins. These conserved proteins may play roles in vital viral functions like DNA replication, immune evasion, and virion assembly [68]. The existence of such proteins in different viral families could be the result of evolutionary convergence in which homologous genes have been retained across different lineages due to similar functional imperatives [69].

The presence of conserved proteins between WSSV and other large dsDNA viruses bears very strong implications for understanding the evolutionary history of WSSV. WSSV also shares several essential genes with viruses infecting different hosts, such as plants and vertebrates, which would imply that these are maintained through evolutionary pressures due to their functionality. This information enhances our understanding of how WSSV may have adapted to its crustacean hosts and developed its pathogenic capabilities. Moreover, the conservation of viral proteins across families has practical applications in the development of antiviral strategies.

### 4.2. Global Genetic Distribution of WSSV (Genome)

The VICTOR program has produced the following neighbor-joining phylogenetic tree (Figure 2), which illustrates the evolutionary relationships between WSSV isolates from a broad geographical range. The tree is rooted by midpoint rooting and displays genetic diversity among the WSSV isolates based on nucleotide sequence similarity. This tree contains high (~100%) bootstrap values for most of the branches, providing very high confidence in clustering, particularly between the more closely related isolates. The isolates from China (NC 075105.1) and Bangladesh (PP134839.1, PP134840.1, PP134841.1) were grouped in one clade, which was strongly supported by a bootstrap value of 100, suggesting an extremely recent common ancestor or a closely related evolutionary origin. Indian isolates, EU327500 and EU327499, and Thai isolates, KX501222.1 and KX501223.1, were phylogenetically tight, indicating regional phylogeographic patterns. This is further reflected in the presence of distant isolates, such as those from Mexico (MG432477.1) and Germany (KF981443.1), outside of primary clusters, indicating significant genetic divergence that might relate to geographical and environmental differences influencing WSSV evolution. Isolates from Saudi Arabia (KF976716.1) and Brazil (HQ130032.1) occupy an intermediate position, with a likely migration or trade-related virus spread. Another distinction involves the groupings from South Korea (GQ328029.1) and Australia (MF161441.1), which, further downstream, split into two lineages diverging from the other Asian core isolates. The branch lengths within the tree themselves are indicative of the mutation rates across the given isolates; some have longer branches, such as Germany and Brazil, indicating higher rates of evolution or separate mutational events.

### 4.3. WSSV Genome Variation and Links to Virulence

Genome architecture, patterns of variation, and pronounced tendency toward genome shrinkage of WSSV have profound implications for virulence and epidemic behavior. The virus is one of the first large invertebrate DNA viruses to have its genome sequenced, with early landmark assemblies reporting complete circular genomes of 292,967 bp (WSSV-CN) and 305,107 bp (WSSV-TW) and identifying ~180–185 ORFs in one assembly and ~181 ORFs in another, establishing that a conserved replication core and essential structural genes (e.g., the major envelope proteins VP28, VP26, VP24 and VP19) are complemented by a large repertoire of accessory and hypothetical ORFs that are often located in repeat-rich, highly variable regions of the genome [70,71]. Comparative genomics across dozens of isolates collected from Asia, the Americas, and Africa over the last two decades has shown that while the core replicative and structural genes are highly conserved, the dominant mode of WSSV genomic plasticity is structural (large deletions and variable-number tandem repeat polymorphisms) rather than simple single-nucleotide substitution, with recurrent deletions mapped to two major variable loci commonly termed the ORF14/15 and ORF23/24 regions and deletion sizes spanning a broad range from a few hundred base pairs up to >20 kb, driving inter-isolate genome size differences often on the order of 10–25 kb [72,73]. Longitudinal and comparative studies provide quantitative examples: in multi-isolate comparisons from China, isolates characterized as high, moderate, and low virulence had reported genome sizes of 309,286 bp, 294,261 bp, and 284,148 bp, respectively, illustrating that genome contraction can be associated with measurable loss of coding potential [72]. Similarly, archival and contemporary sequencing combined with a simple geometric model of deletion accrual found that some regions, particularly the ORF23/24 locus, show a statistically significant relationship between deletion size and time since the first outbreak in a country and parameter estimates consistent with a geometric progression of genomic deletions during epidemic spread [74]. Mechanistically, these recurrent deletions appear to arise from recombination between direct repeats and from structural instability in repeat-rich regions; the sequencing of multiple isolates with long-read and short-read data has revealed complex junctions at deletion breakpoints consistent with non-homologous recombination and repeat-mediated rearrangement, and the presence of host-derived or virus-derived repeated elements complicates assemblies, sometimes producing apparent size variation that must be validated with long reads or PCR across junctions [75,76]. From an evolutionary standpoint, genome shrinkage in WSSV appears to be real and systematic in many lineages rather than merely an assembly artifact; empirical data compiled from multi-country surveillance cohorts indicate that some epidemic lineages show cumulative loss of accessory genes over serial outbreaks, resulting in net genome contraction on the order of ~5–20 kb over periods of years to decades, and modeling work suggests the rate of shrinkage decelerates over time as the pool of easily lost accessory loci is exhausted [74]. The adaptive significance of shrinkage is debated but plausible mechanisms include selection for replication economy in high-density aquaculture (where faster replication or reduced genomic maintenance costs could confer competitive advantages), genetic drift and repeated founder events during anthropogenic translocations, and molecular mechanisms such as recombination at repeat motifs that produce deletion variants; importantly, endemic host factors such as endogenous viral elements (EVEs) present in shrimp genomes may influence observed viral sequence diversity by providing homologous regions for recombination or by priming host RNAi responses that select for particular viral genotypes [1,76]. Turning to phenotype, multiple controlled infection experiments and comparative pathogenicity assays have examined how genome architecture maps onto virulence (lethality) and, far less commonly, transmissibility; a consistent pattern emerging from this corpus is that isolates with extensive deletions or reduced genome sizes often show attenuated lethality in laboratory bioassays, typically reported as increased LD_50_ values, delayed time to death, or reduced cumulative mortality relative to full-length or ancestral isolates findings that have been reported in experimental comparisons in which isolates carrying deletions >10 kb displayed LD_50_ values one to two orders of magnitude higher and median survival times extended by 24–72 h in *Penaeus* spp. under comparable challenge conditions [72,77]. However, the virulence signal is far from absolute; host species, life stage, infection route (intramuscular injection vs. immersion), environmental variables (temperature, salinity), and infectious dose all interact to determine disease outcomes, such that some “attenuated” deletion-bearing isolates still produce high (>70–90%) mortality when delivered by immersion at high doses to susceptible juveniles, indicating that the observed attenuation often manifests as a shift in dose–response rather than complete loss of pathogenicity [78,79]. The relationship between genomic variants and transmissibility is more ambiguous; relatively few studies measure direct transmission metrics (e.g., viral shedding rate, environmental persistence, R_0_ in pond or experimental cohabitation models) in the same experiments that measure lethality, but the limited data show heterogeneous results, some deletion variants with lower per-host lethality shed comparable viral loads and sustain waterborne transmission in cohabitation assays (creating the theoretical possibility that lower lethality extends host infectious periods and thereby increases cumulative transmission), whereas other shrunken genomes produce lower viral titers and reduced secondary infection in sentinel animals, demonstrating that transmissibility is not rigidly predictable from genome size alone [80]. At the population and epidemiological levels, phylogeographic reconstructions and outbreak tracing consistently show rapid global dissemination of WSSV lineages that aligns strongly with shrimp trade routes, broodstock movement, and frozen shrimp imports rather than with gradual, within-region selection for a hyper-transmissible genotype; indeed, the initial introductions of WSSV into the Americas in the late 1990s and subsequent spread across regions correspond temporally and geographically with commercial movements, and genotype turnover in many countries is best explained by repeated introduction events and founder effects rather than by local selective sweeps [13,81].

### 4.4. Transmission Dynamics of WSSV

The WSSV is the most virulent pathogen affecting global shrimp aquaculture, and unraveling its dynamics of transmission is crucial for effective mitigation strategies. WSSV is a highly contagious, lethal, double-stranded DNA virus of the *Nimaviridae* family that often causes large-scale mortalities [13]. It has vertical and horizontal routes of transmission, both of which contribute to the rapid spread of the disease in different shrimp farms and even in nature. Horizontal transmission is the more common mode of transmission; this includes direct contamination through waterborne contact, infected shrimp, and organic materials such as feces and molts [79,82]. Waterborne transmission is most important because infected shrimp release viral particles into the water through gill shedding, from body surfaces or during decomposition, hence producing a heavily contaminated milieu. It has been observed that even an extremely low level of virus-contaminated water may mediate the spread of WSSV, and viral shedding can be detectable within hours of infection onset [82]. This environmental transmission is a serious concern in densely populated shrimp farming systems where high stocking densities enhance the risk of infection. In infected shrimp, WSSV advances with rapid and lethal progression.

Significantly, it has been found that compared to the important transmission route of cannibalism or ingestion of infected tissues, waterborne exposure poses a greater infection risk in high-density farming [1,83]. Clearly, this makes the design of biosecurity protocols very relevant; it implies that control over water quality and reduction in waterborne exposures should be emphasized above preventing cannibalism. In addition to these pathways, live feed organisms such as copepods and rotifers have been implicated as WSSV carriers capable of transmitting the virus directly to shrimp during feeding. For instance, copepods infected with WSSV have demonstrated infectivity in challenge experiments [84], and rotifers (*Brachionus plicatilis*) caused up to 82% mortality in *P. monodon* post-larvae [85]. While these findings are indicative of a putative mode of transmission, especially in hatchery environments where such feeds are common, their epidemiological significance in commercial farming conditions is questionable. Subsequent studies reveal that aquaculture relying on zooplankton-enriched live feeds (e.g., aquamimicry) could perpetuate viral circulation if due precautions are not taken [86]. Furthermore, a systematic review reaffirmed that zooplankton, such as copepods, are part of the complex WSSV host–vector network, although field observations remain scarce [80]. While this route is plausible in hatchery settings, its impact under farm conditions remains uncertain and likely context-dependent. Nevertheless, proper disinfection of live feeds is advisable to limit risk. On the other hand, vertical transmission becomes evident when the virus-carrying broodstock is used to transmit the virus to progeny through spawning [87]. This has been considered a very injurious route of infection in hatcheries since asymptomatic carriers can spread the virus unknowingly to those populations.

**Figure 3:**
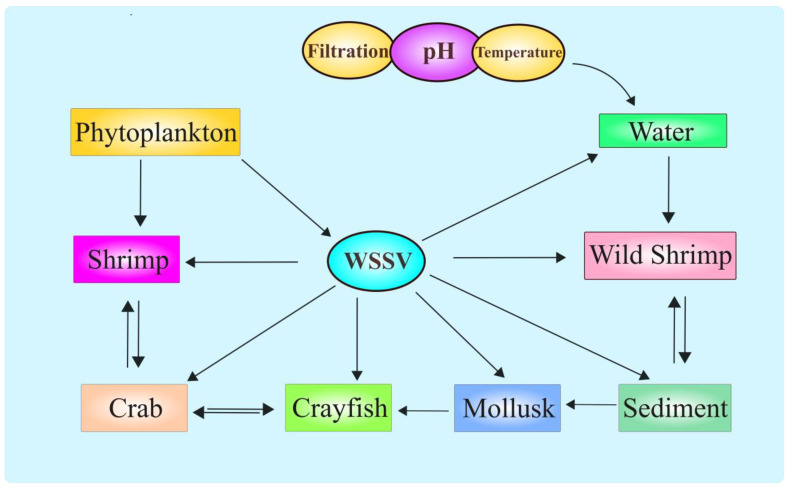
Transmission pathways and influencing factors for White Spot Syndrome Virus (WSSV) in shrimp aquaculture.

WSSV does not exclusively affect shrimp and can affect a wide variety of crustacean and non-crustacean species as carriers and vectors both in aquaculture and the wild [88,89,90,91]. For instance, WSSV was isolated from crabs [92], crayfish [93], and other decapods [94] (Figure 3), broadening the circle of potential inter-species infection and increasing the degree of difficulty in containment measures. These findings further stress the generalist nature of WSSV, able to thrive under varying conditions of brackish and freshwater systems. Such adaptability carries further control complications, especially in open systems where farmed and wild populations interact. One of the main stumbling blocks in interpreting the transmission routes of WSSV is likely to be the variation in impact due to different environmental factors like temperature, salinity, and pH [95]. Studies have demonstrated that the rate of virus replication as well as the speed of diffusion are higher at elevated temperatures, while variation in salinity can lead to differences in susceptibility among shrimp to the virus [12]. For example, in tropical regions, where temperatures are consistently high, WSSV outbreaks are more serious with rapid disease development and higher mortalities. Moreover, the virus appears to be stable within a broad range of salinity levels, which allows it to infect shrimp in both marine and freshwater aquaculture.

However, despite advances in understanding WSSV transmission dynamics, substantial knowledge gaps still remain, particularly relating to the role of non-crustacean species and environmental reservoirs in the perpetuation of the virus. Furthermore, the presence of wild species that could be carriers or reservoirs of WSSV complicates efforts to establish WSSV-free zones in aquaculture. Additionally, the persistence of the virus in the environment, even in the absence of host species, raises concerns about the long-term sustainability of shrimp farming in such locations [4]. One proposed solution has been compartmentalization farming, a concept where shrimp are reared in biosecure units, inhibiting the spread of pathogens at both farm and external environment levels [96]. These techniques have achieved limited success, and the high cost of retrofitting has precluded widespread adoption. Future research should focus on elucidating the precise mechanisms of WSSV transmission in mixed-species environments, as well as developing novel strategies for disease prevention and control. With such diverse routes of virus transmission, further investigation into the host range, whether through natural or experimental infection and together with the interaction of host proteins with the virus during replication and dissemination, will clarify the complex epidemiology of WSSV.

### 4.5. Host Species Reported to Be Naturally or Experimentally with WSSV

A wide range of hosts, including economically important different shrimp species and organisms from both fresh and marine environments, have been found to be infected with viral pathogens. For WSSV, hosts in a wide array of shrimp species have been detected from *Penaeus monodon* [36], *Penaeus vannamei* [97], *Marsupenaeus japonicus* [98], and *Penaeus chinensis* [99]. In addition to these economically valuable penaeid species, WSSV has been isolated from crabs (*Scylla olivacea*, *Neohelice granulate*) [92], copepods [100], lobsters [101], crayfish, i.e., *Procambarus clarkii* [102], and freshwater species such as *Macrobrachium rosenbergii* [91]. Furthermore, it was confirmed that WSSV primarily afflicts decapod crustaceans, but recent studies demonstrate that its host range is further expanding. A study by Desrina et al. emphasized that WSSV infects species from more than 50 families, including non-crustacean hosts such as mollusks, though crustaceans remain the primary hosts [103]. In experimental conditions, the transmission and replication of WSSV have been confirmed in non-target species like *Metapenaeus ensis* [104], *Exopalaemon orientalis*, and *Calappa lophos* [83], where non-target species might act as reservoirs or vectors (for more details, see Table 2). The wide host range of WSSV, comprising different species of shrimps and other crustaceans, is partly due to the ability of WSSV to bind a wide variety of host proteins that are conserved or functionally similar across hosts.

### 4.6. WSSV Virion Proteins

The WSSV virion is an extremely infective particle and thus very important in the process of disease transmission [68]. Structurally, this virion is a rod-shaped enveloped, non-occluded particle composed of macromolecules arranged to protect and convey the viral genome to effect infection in host organisms. The width of the WSSV virion ranges from 70 to 170 nm, while the length falls between 210 and 420 nm. The virion is composed of three layers: the tegument layer, the envelope, and the nucleocapsid (300 by 70 nm and enveloped by a layer of capsids) [8]. Each of these layers play a role in the integrity and infectivity of the virus. WSSV has at least 58 structural proteins, of which localization data is available for 48 [107]. Among these, 33 are envelope proteins, 9 are nucleocapsid proteins, and 5 are tegument proteins [8]. Of these envelope proteins, the major ones are VP28 and VP26 proteins, making up about 60% of the envelope proteins. The envelope proteins play an important part in WSSV’s infectivity by means of binding. VP28 is a well-known protein that handles cell surface recognition as a receptor for the virus to attach to the host cell membrane [71,108,109]. This mechanism of binding is critical for successful infection in shrimp because it allows the virus to pass into the cytoplasm of the host cell. Thus, VP28 may be considered a prime target for antiviral treatment because inhibiting this protein is normally adequate to block the virus from attaching to or entering host cells.

Besides VP28, other envelope proteins VP31, VP33, VP36A, VP110, VP136A, and VP664 contain cell attachment motifs that may facilitate the initial stage of viral infection [109,110,111]. These motifs allow the virus to attach to the host cell surface, making them an important feature for the development of therapeutic strategies. Among them, VP664 is one of the most abundant and largest proteins, comprising 6077 amino acids, and plays a very important role in viral replication. Interfering with the functioning of VP664 might disturb replication of the virus and potentially offer another avenue for pharmaceutical intervention. A more detailed breakdown of the structural proteins (VP28, VP39B, VP31A, VP41B, VP51A, VP51B, VP68, VP124, VP150, VP187, VP281, and VP292) found in the WSSV envelope [107] and other proteins (VP190, VP466, VP15, VP51, and VP76) derived from a collagen-like protein in the nucleocapsid of WSSV [112] are listed in Table 3 according to their respective locations in the virion: envelope, tegument, or nucleocapsid. For instance, for proteins such as VP124, VP187, and VP466, their kDa and ORF values reflect their capability to enhance the WSSV virion’s invasiveness into host cells; thus, further understanding of their structure and function could eventually facilitate targeted treatments [111].

Homologous relationship of WSSV proteins with other viral proteins provides further potential targets that could be used in drug development. For instance, proteins such as VP26 and VP28 have roles in maintaining the structural integrity of the virion and are also involved in a series of steps which result in infection [115,116]. Small molecules or peptides could be designed to interfere with the structural roles of these proteins, preventing proper virion assembly or entry into host cells [117]. These can be targeted therapeutically by devising means through which the functions of these proteins are disrupted to inhibit the virus from spreading. Another idea that might be significant is that proteins with glutathione S-transferase fusion, like ORF151-VP466, can be targets that allow improvements in the host immune response, or inhibit viral processes [118]. Indeed, these key identifications allow the possibility of developing vaccines or antiviral medications targeting the virus life cycle at points intended to interfere with infecting and replicating within shrimp, thereby reducing aquaculture losses attributed to WSSV.

### 4.7. Molecular Mechanisms of WSSV Life Cycle: Host Protein Contributions

**Figure 4:**
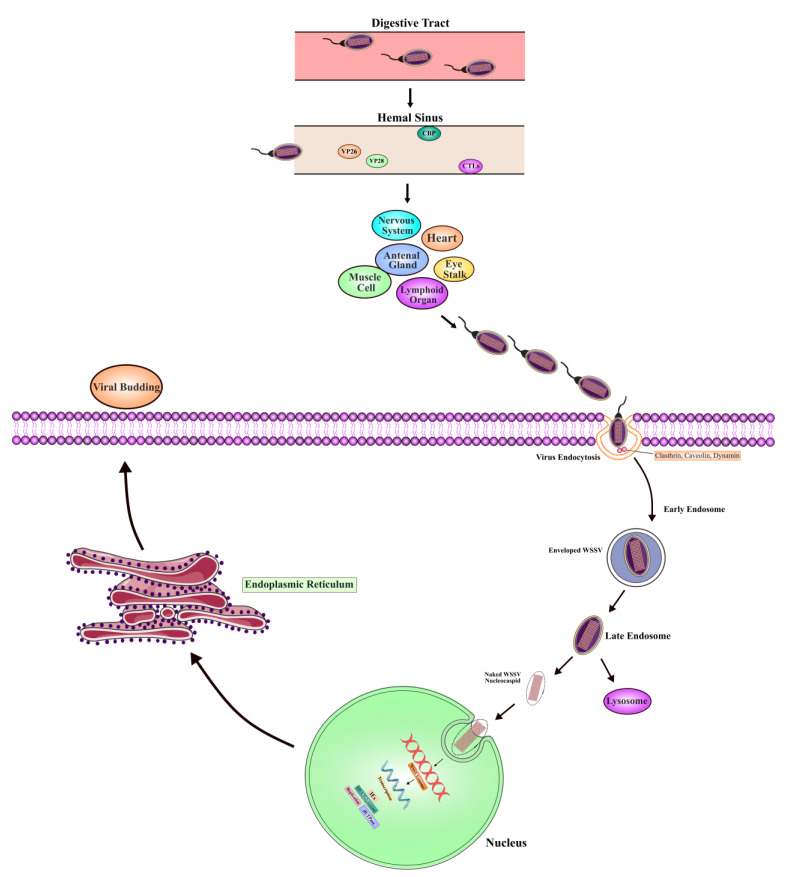
Life cycle of WSSV in shrimp illustrating viral entry through the digestive tract, dissemination via hemal sinuses, receptor-mediated endocytosis into target cells, replication of the viral genome within the nucleus, and subsequent release of mature virions through the endoplasmic reticulum and plasma membrane.

The molecular underpinning of the life cycle mechanisms involves an elaborate interplay between viral components and host cell machinery (Figure 4). Interactions of host proteins with viral proteins at many steps in the infection process are indeed critical to the successful replication and spread of the virus through the host organism. These proteins facilitate not only the entry of the virus into the host cell but also contribute to intracellular trafficking, viral replication, assembly, and egress. Here, we have focused on the participation of host proteins in each critical stage of the WSSV life cycle from viral entry to progeny virion release.

Entry of viruses into host cells: The process of infection is initiated when WSSV virions come into contact with host cells through the ingestion of infected or dead shrimp. Infection is mainly via the digestive tract, where the viral particles come into contact with and attach to the receptors of the host cells lining the epithelium (Figure 4). These interactions are mediated by host proteins serving as receptors/co-receptors for the virus, which allow viral attachment to the surface of the cell. Several host receptors have been identified as enhancers of viral infection and facilitators of viral passage across the basal membrane into the circulatory system, ultimately reaching target organs. These include glucose transporter 1 (Glut1), C-type lectins (CLs), chitin-binding proteins (CBPs), peritrophin-like proteins (PTs), tetraspanins, cuticle proteins, and thrombospondins [119,120,121,122,123,124]. In this regard, one of the most studied receptor families involves the CBPs, more specifically the PmCBP in *Penaeus monodon*, which is a critical participant in mediating WSSV attachment. Hence, this protein is capable of binding at least 11 envelope proteins of WSSV and establishes a stable interaction through which the virus can initiate entry into the host cell, such as VP24, VP32, VP39B, VP41A, VP51B, VP53A, and VP110 [119]. Moreover, PTs play a vital role in the gut as receptors, especially in *L. vannamei*, where LvPT interacts with viral proteins like VP32, VP38A, and VP39B [125]. These proteins are secreted into the stomach, possess potent chitin binding properties, and help in the transportation of viral particles across the epithelium of the digestive tract. Bound to these receptors, the WSSV particles penetrate the epithelial cells, cross over the basal membrane, and enter further into the circulatory system. Another major molecule that plays an important role in viral entry is the glucose transporter 1 (Glut1) protein, expressed in almost all tissues including the digestive tract, muscles, and pleopods. Glut1 plays a complementary role in identifying several envelope proteins of WSSV, such as VP28 and VP53A, during viral entry into cells [120]. This protein binds to at least seven viral envelope proteins in an adjoining loop region, making it a key mediator of WSSV infection. More recent studies have proposed a complex of Glut1 forming with PmCBP, presenting a larger surface area to the virus by making viral binding and, therefore, attachment and internalization more effective [126]. Tetraspanins are transmembrane proteins that bridge connecting inner and outer cell membrane proteins, which serve as a vital group of receptors for WSSV. Structurally, it has four transmembrane segments with small and large extracellular loops. These proteins include notable types in shrimp such as FcTetraspanin-3, FcCD63, and FcCD9. Among them, FcTetraspanin-3 is thought to play a crucial role in responding to WSSV infection [127]. Conversely, large extracellular loops inhibit the FcTetraspanin-3 domain to WSSV infection [128]. Shrimp have an open vascular system, allowing soluble proteins to spread across the whole body through the circulating plasma. C-type lectin (CL) was mainly identified in the stomach and muscle of shrimp. Once viral protein and CL complex are bound to each other, they are then secreted into the circulating system after the complex spreads throughout the whole body by the hemolymph, which provokes a systemic infection. In shrimp, CL has been demonstrated to interact with several WSSV proteins including LvCTL1 (*L. vannamei*) that can interact with VP14, VP24, VP28, VP26, and VP95. Along with *F. chinensis* (FcLec3), lectins that can interact with VP28 [129] were identified. In *M. japanicus*, three lectins and one c-type lectin, e.g., MjLecA, MjlecB, MjlecC, and MjsvCL were identified to interact with the viral proteins such as VP26, VP28, and VP281 [130]. After WSSV viral proteins enter the Hemal sinuses, penetrating the digestive tract, the virus reaches different body compartments throughout the hemolymph circulation. In this stage, Integrin proteins provide essential receptors for viral infection to target organ cell surfaces (gonads, heart, eyestalk, muscle, nervous system cells, and hematopoietic cells). The integrin receptor of *L. vannamei* was identified to bind with viral proteins VP26, VP31, VP37, VP90, and VP136. These interactions predominantly occur through specific motifs, including RGD, YGL, and LDV [125]. After binding to the receptors on the target organ cell surface, the enveloped virus can either penetrate the membrane directly or undergo uptake through endocytosis [131].

Endocytosis and intracellular trafficking: Attached to the host cell, the main mode of WSSV entry into the cell is through receptor-mediated endocytosis. This allows the virus to bypass the host cell membrane into the intracellular environment. So far, the best-characterized pathway of endocytosis exploited by WSSV is clathrin-mediated endocytosis, where the virus is engulfed into clathrin-coated vesicles that bud off from the plasma membrane [132]. These vesicles ferry the virus to early endosomes, in which the low pH allows viral uncoating to occur. This acidic environment is essential for initiating conformational changes in the virus, leading to the release of the viral genome and nucleocapsid into the host cytoplasm [131]. Also, endocytosis depends on cholesterol and dynamin, evidenced by studies showing that WSSV invasion depends on these lipid components for membrane curvature and vesicle scission [133]. The virus avoids lysosomal degradation via Rab GTPases, which regulate membrane trafficking events during endocytosis. Specifically, Rab5, during development in *P. monodon* and *L. vannamei*, mediates the maturation of early endosomes into late endosomes. Rab7 is involved in the later stages of endosome maturation and replaces Rab5, ensuring transport of viral nucleocapsid to the host cell nucleus without degradation [134].

Viral genome delivery and replication: Once inside the host’s cytoplasm, the viral nucleocapsid has to reach the nucleus, where replication occurs. The viral envelope fuses with the host endosomal membrane and discharges the nucleocapsid into the cytoplasm, which then migrates to the nucleus. The nuclear pore complexes transport the viral genome into the nucleus. This marks the start of the replication phase. Inside the nucleus, WSSV expresses its immediate early genes, which are considered crucial for initiating the replication machinery. These early genes allow for the synthesis of mRNA, which is transported back to the cytoplasm for translation. The viral mRNA then undergoes translation in the cytoplasm by free ribosomes, encoding a major structural protein, VP664, which forms the backbone of the WSSV capsid. This occurs in parallel with the replication of the viral genome and the synthesis of viral proteins, enabling the assembly of new viral particles [110]. The involvement of host proteins continues to play a critical role in these processes. For instance, Glut1 mediates the trafficking of viral proteins between cellular compartments, while the tetraspanins like FcTetraspanin-3 bridge the connection between the inner and outer cell membranes to help mediate the intracellular locomotion of viral components [127,128].

Viral assembly and egress: Assembly of the virus occurs near the host nucleus, where the major capsid proteins, including VP664, form the new structure of virions. These assembled virions incorporate viral envelope proteins like VP28, synthesized in the rough endoplasmic reticulum. These envelope proteins are then targeted to the inner nuclear membrane, where they associate with the assembling virions. VP28 is an essential protein for infection efficiency and has been shown to stabilize the viral envelope and increase the infectivity of the virions [135]. As the viral load within the host cell increases, it builds up until the accumulation of viral particles overwhelms the cell. Finally, lysis of the host cell releases newly formed virions into the extracellular environment. Completion of the WSSV life cycle enables the virus to infect neighboring cells and to spread throughout the host organism. The lysis of infected cells is a pivotal event that permits the rapid propagation underlying the systemic infection that is typical of WSSV.

Systemic spread and organ targeting: Once internalized into the hemolymph, the virus is circulated via the open vascular system of the host to infect a wide array of tissues and organs of mesodermal and ectodermal origin. Integrin proteins on the surface of target cells, including those of the gonads, heart, muscles, and nervous system, constitute important docking points for WSSV. Integrins in *L. vannamei* bind viral proteins VP26, VP31, VP37, VP90, and VP136, thereby facilitating virus attachment and penetration of these vital tissues [125]. There exist specific viral motifs, such as RGD, YGL, and LDV, which enable the virus to recognize and bind integrins on the host cell surface through such interactions. The virus can either penetrate directly into the host cell membrane once bound to the integrins or be internalized by receptor-mediated endocytosis. WSSV research has shown that both clathrin-mediated and caveolae-mediated pathways are used for endocytosis depending on the host cell and tissue type [110]. Caveolae-mediated endocytosis is particularly cholesterol-dependent, and it promotes internalization within vesicles, thus bypassing lysosomal degradation of viral particles. The systemic spread of WSSV within the host is further facilitated by CLs that bind viral proteins and facilitate the transportation of the virus through the circulatory system [136]. In *L. vannamei*, LvCTL1 binds to viral proteins VP14, VP24, VP28, and VP95 and helps distribute them in the body via the hemolymph. These interactions initiate a systemic infection, with the virus targeting multiple organs and tissues, including hemal sinuses, gonads, and eyestalk [129].

Host defense evasion mechanisms: WSSV expresses several mechanisms that enable the pathogen to evade host immune responses to establish a productive infection. This includes manipulation of host endosomal trafficking pathways to avoid degradation by lysosomes, in which the Rab GTPases, notably Rab5 and Rab7, play important roles [1]. Rab5 controls endosomal maturation, thereby facilitating transport of viral nucleocapsids from early to late endosomes. Rab7 ensures that the viral particles are not targeted to lysosomes for degradation [134]. In this way, the virus evades host immune responses and possibly persists in a latent state for longer periods. WSSV also manipulates immunomodulation within its host by interacting with immune-related proteins, such as tetraspanins and lectins, which act as receptors with important implications in immune signaling. The virus, through binding, may alter the immune response and inhibit processes leading to the production of antiviral mechanisms, thereby enhancing its survival and replication within the host.

## 5. Pathology

### 5.1. Gross Sign of WSSV

Gross clinical signs in animals infected with WSSV vary considerably at different stages of the infection process; however, white spots on the cuticle are pathognomonic signs for this viral disease in some shrimp species. These appear as white spots, 1–3 mm in diameter, and are calcium-rich viral accumulations on the exoskeleton especially on the cephalothorax, appendages, and abdomen [68]. White spots do not occur in all WSSV infections, and identical lesions due to non-viral etiology such as bacterial infection or shell mineralization disease are also possible, thereby making them an unsatisfactory single diagnostic feature. In species such as the *Penaeus monodon*, *Penaeus vannamei*, and *Marsupenaeus japonicus*, infected individuals, in general, become lethargic, exhibiting decreased feeding and erratic swimming before eventually succumbing to the disease [11]. Infected shrimp usually move to the edge of the ponds or to the water surface, where they are preyed upon by predatory birds that can facilitate mechanical transmission of the virus [137]. Characteristic symptoms of infection vary from species to species which include soft shells, discoloration of the body to reddish, and loose appendages in worst cases (Table 4). Similar signs are shown by *Penaeus chinensis*, with additional symptoms of soft bodies and gill necrosis [101]. The signs of WSSV infection in *Macrobrachium rosenbergii* (giant freshwater prawn) include red bodies, gill necrosis, and lethargy [138]. Experimental studies have shown that infected shrimp can begin shedding viral DNA into the water within six hours of infection, with shedding peaking just before death. This greatly elevates the risk of infection to surrounding shrimp, particularly in closed systems like grow-out ponds [80,139,140]. Other susceptible decapod crustaceans, like *Metapenaeus ensis* and *Exopalaemon orientalis*, also exhibit similar clinical manifestations of incapacitated mobility, reddening of the body, and lesions in the exoskeleton. WSSV is histologically characterized as an ectodermal and mesodermal tissue-attacking virus. Very severe degenerations are shown among infected gills, lymphoid organs, and antennal glands [83]. WSSV infection in *Scylla olivacea* and *Neohelice granulata* causes lethargy, white spots on the carapace, and internal tissue necrosis [141]. The same type of white spots develop on lobsters and crayfish (*Procambarus clarkia*), infected with WSSV, along with erratic swimming, often accompanied by discolored or darkened exoskeletons [142]. Even copepods and mollusks, which are less infected, may act as passive carriers or mechanical vectors of WSSV, especially after being exposed to high viral loads from contaminated environments [100]. Although such organisms may occasionally harbor viral particles, the current evidence does not show that they allow productive viral replication, and therefore function primarily to propagate transmission but not disease expression [143]. WSSV exerts its pathogenicity towards host species by virtue of tissue tropism for vital tissues such as cuticle and haemopoietic systems, inducing degeneration and death. Poor water quality may act as an environmental stressor that enhances disease progression through increased susceptibility and more rapid viral replication. In addition, certain factors such as rising or fluctuating temperature, salinity imbalance, and high stocking density are also found to impair the immune response of the shrimp, contributing to the progression and severity of WSSV outbreaks [12]. Behavioral changes, including convulsions and reduced mobility, can be observed in the late stages of infection, often in severely diseased populations.

### 5.2. Histopathology of WSSV

**Figure 5:**
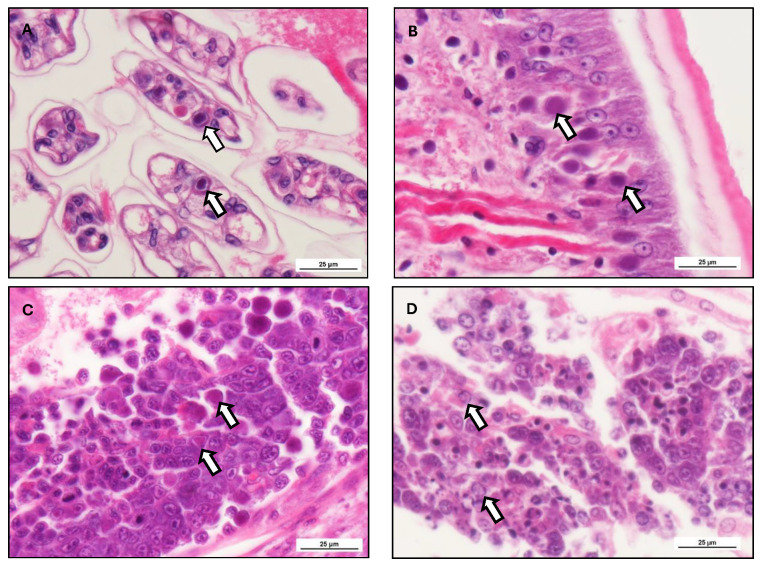
White Spot Syndrome Virus (WSSV) within *Penaeus monodon* tissues. (**A**) WSSV in gill tissues; enlarged nuclei with marginalized chromatin and containing eosinophilic inclusions (arrows) can be observed distributed throughout the cuticular epithelium of the gill filaments of an infected shrimp. (**B**) Cuticular epithelial cells of the stomach showing hypertrophied nuclei with eosinophilic staining (arrows). (**C**) WSSV infection within the haematopoietic tissue. Affected cells displaying enlarged nuclei with eosinophilic inclusions (arrows). (**D**) WSSV-infected lymphoid organ tissues displaying loss of structure of tubules. Enlarged nuclei with marginalized chromatin can be seen throughout (arrows). Lymphoid organ spherules will develop as tissue structure is lost. All images H&E Stain. Scale bars = 25 µm.

The histopathological changes in WSSV reflect the pathogenesis, disease course, and immunity associated with WSSV (Figure 5). The main target cells of WSSV are the ectodermal and mesodermal tissues [144], including the cuticular epithelium, gills, lymphoid organ, foregut. In the hepatopancreas, WSSV infection has been observed predominantly in the haemocytes and connective tissues surrounding the tubules, but not in tubular epithelial cells themselves [20]. The representation of WSSV infection features hypertrophied nuclei with intranuclear inclusion bodies of a basophilic nature, pyknosis, karyorrhexis, and cytoplasmic vacuolization [44]. As infection progresses, these cellular changes result in widespread necrosis with attendant impaired physiologic function of infected organs (organ specific information available in Table 5). Gill tissues exhibit epithelial sloughing and lamellar fusion, severely reducing respiratory efficiency, and the hepatopancreas, a principal organ of shrimp metabolism, degenerates, often complicated by secondary bacterial infections [145]. The lymphoid organ plays a critical role in immunity as well, but in WSSV-infected shrimp, it undergoes lymphoid organ spheroid (LOS) formation, which is an attempt to limit viral replication, but is unsuccessful because viral replication happens so rapidly [146]. Transmission electron microscopy (TEM) has revealed that WSSV virions are rod-like, approximately 275 nm in length and 120 nm in diameter [135], enveloped with a double-stranded DNA genome in a lipid envelope. Virus replication occurs in the nuclei of infected epithelial cells [147]. Compared to other viruses of shrimp such as Infectious Hypodermal and Hematopoietic Necrosis Virus and Taura Syndrome Virus, which essentially infect haemocytes, WSSV has a tropism for epithelial tissues that significantly aggravates systemic infections [68]. The progression of WSSV infection is inconsistent, taking either acute or chronic forms. From histopathological observation, it has also been ascertained that massive tissue necrosis, intense virus replication, and extensive intranuclear inclusions happen in acute WSSV infections [148], resulting in explosive mortality of shrimp. In contrast, chronic infections consist of persistent, low-grade viral replication, with localized tissue damage and immune suppression, resulting in growth impairment and increased susceptibility to secondary infections.

Histopathological grading is also an important technique for ascertaining WSSV severity and evaluating the extent of disease progression. The intensity of infection is classified into four grades: G0 (no infection), G1 (mild infection with nuclear hypertrophy in fewer than 10% of cells), G2 (moderate infection with inclusion bodies in 30–50% of infected cells and mild necrosis), G3 (severe infection with inclusion bodies in more than 50% of cells, together with extensive necrosis), and G4 (late infection with complete cell destruction in multiple organs) [82,154]. Molecular diagnostic techniques such as in situ hybridization (ISH) and polymerase chain reaction (PCR) significantly enhance the sensitivity of WSSV detection. Parallel to ISH, normal histology and electron microscopy permit precise localization of viral DNA in infected tissues, which usually reveals strong signals in connective tissue, gill lamellae, and reproductive organs, suggesting potential vertical transmission [83,155]. Histopathology remains a vital diagnostic tool for early detection of the disease, enforcement of biosecurity, and treatment evaluation. Preventive strategies such as probiotics, immunostimulants, and plant extracts have shown promise in reducing histopathological damage and improving survival rates in shrimp [156]. Additionally, histological examination is employed to examine the efficacy of antiviral drugs in managing WSSV-caused tissue pathology [157]. The combination of histopathology and molecular diagnosis is more effective at disease detection and control. It is noteworthy that histopathological lesion provides a presumptive diagnosis of WSSV. In accordance with the World Organization for Animal Health diagnostic manual, confirmatory diagnosis is required to be carried out with PCR and better sequencing to ascertain the presence of WSSV-specific genomic material, which is very much essential for accurate reporting and disease surveillance in accordance with international standards.

### 5.3. Co-Infection of WSSV and Other Disease of Shrimp

The prevalence of disease pattern in shrimp farms has shifted from, previously, simple infection to co-infection including complicated infections, mixed infections, super infections, polymicrobial diseases, secondary infections, multiple infections, dual infections, and concurrent infections of homologous or heterologous pathogens in recent years [158,159,160]. Simultaneous or sequential co-exposure of heterologous pathogen (parasite-bacteria, parasite-virus, virus-bacteria, fungus-bacteria), also termed as co-infection, is further defined as the infection of host animal (shrimp) from two or more genetically distinct pathogens, each of which has pathogenic effects and harms the host in concert with other infections. Among different patterns of pathogen exposure to shrimp species (for details see Table 6), homologous combinations of viruses (WSSV, Taura syndrome virus (TSV), hepatopancreatic parvovirus and infectious myonecrosis virus (IMNV), infectious hypodermal and haematopoietic necrosis virus (IHHNV), monodon baculovirus (MBV)) [29,150,161,162,163] and bacteria (*Bacillus cereus*, *Bacillus flexus*, *Shewanella decolorationis*, *Aeromonas veronii*, *Shewanella amazonensis* and *Kurthia gibsonii*) are most extensively investigated [164].

For shrimp species, parasite–virus co-infections are relatively uncommon and were first reported involving a microsporidian parasite (*Enterocytozoon hepatopenaei*) and viral pathogens (Taura syndrome virus and infectious myonecrosis virus) [180,182]. It is now increasingly evident that EHP has become a substantial global threat to shrimp aquaculture with the economic loss from this microsporidian being approximately double that of WSSV for the Indian shrimp industry in 2021 [28]. Moreover, co-infection of EHP and WSSV [178] from synergistic interaction between these genetically distinct pathogens has raised greater concern regarding the transmission dynamics of microsporidians in shrimp farming systems and adjacent ecosystems, which is an emerging threat to the shrimp industry in Bangladesh.

## 6. Immunological Responses of Shrimp to WSSV

Compared to the possession of real adaptive immune system in vertebrates, shrimp have a well-developed innate defense mechanism. The system is characterized by a non-specific immunological response, usually segregated into cellular and humoral components and activated pattern recognition receptors (PRPs) [183]. So far, several PRRs have been identified in *Penaeid* shrimp including toll-like receptors (TLRs), lectin, tetraspanin, and lipopolysaccharide and β-1,3-glucan binding protein [184]. These receptors play a central role in the recognition of pathogens and in triggering of immune responses in shrimp.

**Figure 6:**
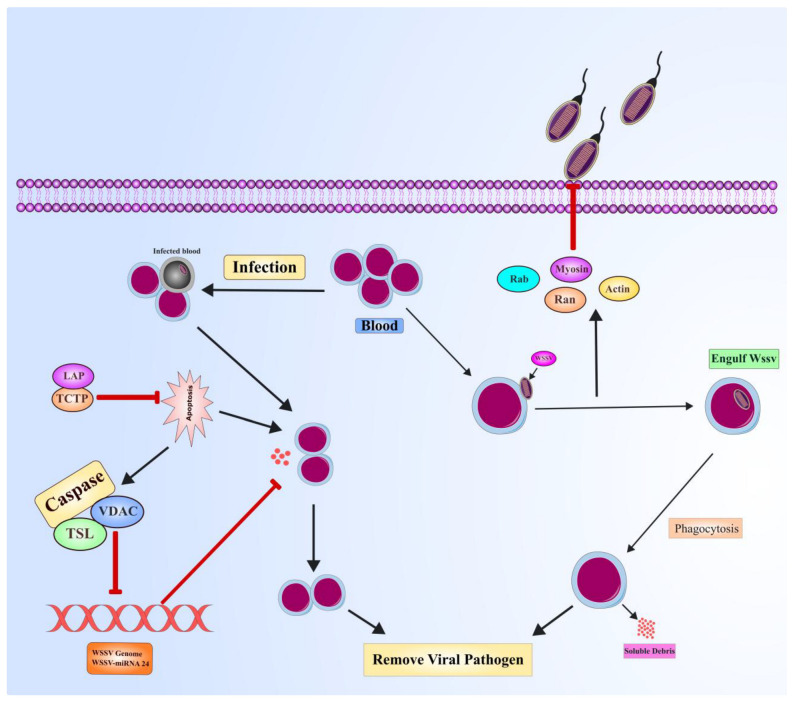
Cellular immune response mechanisms in shrimp against White Spot Syndrome Virus (WSSV) infection.

Cellular immune mechanisms in shrimp: Cellular immunity addresses the recognition and elimination of pathogens through various mechanisms, including phagocytosis, encapsulation, and apoptosis (Figure 6). Hemocytes are circulating immune cells that essentially carry out the process in shrimp through phagocytosis, wherein pathogens are taken up and degraded. This also involves different small GTP-binding proteins such as Ran and Rab [185], reflecting the complexity of shrimp cellular immune responses. Crucially, hemocytes depend on immune mediators like lectins to improve their ability to recognize and respond to pathogens. Lectins are one of the most common classes of immune mediators, which are characterized by a carbohydrate recognition domain [186]. In shrimp, C-type lectins (CTLs) facilitate phagocytosis of microbial pathogens through opsonization, marking the pathogens for ingestion by immune cells. More directly, CTLs exhibit immunity by agglutination and inhibiting microbial growth, including gram-positive and gram-negative bacteria [187]. C-type lectins also show antiviral activity, especially against WSSV. A range of lactins, such as MjsvCL, LdlrLec1, LdlrLec2, LvAV, FmLC5, and FLdlr (found in *Fenneropenaeus merguiensis*, *Litopenaeus vannamei*, and *Marsupenaeus japonicus* species) have been reported with anti-WSSV functions [188]. However, some lectin genes, for example, LvCTL3 (found in *L. vannamei*) and FmLC3 (found in *F. merguiensis*), are paradoxically demonstrated to enhance their vulnerability to WSSV [189]. These findings demonstrate the complexity of lectin immune modulation wherein their regulatory role may support or hinder resistance against pathogens. Recent studies have shown that the NFkB pathway could modulate CTLs expression in *L. vannamei*. [190], again suggesting that in shrimp, immune responses are controlled at every level in a highly ordered way.

Programmed cell death or apoptosis is another aspect of cellular immunity that helps shrimp eliminate cells harboring infectious agents [191]. Apoptosis is typically brought about by various apoptosis-related genes such as caspases, inhibitor of apoptosis protein (IAP), apoptosis-inducing factor (AIF), cytochrome c, the mitochondrial voltage-dependent anion channel (VDAC), Fortilin or translationally controlled tumor protein (TCTP), gC1qR, BAX inhibitor-1 (BI-1), and apoptosis signal-regulating kinase 1 (ASK1) [192]. These molecular components are important in coordinating the controlled and targeted destruction of cells, which is an important feature of the shrimp’s immune response [193]. Caspase are proteases that initiate the early phase of apoptosis in response to external signals. Apoptosis allows shrimp to limit pathogen dissemination in tissues by destroying infected cells in a timely manner [194]. Shrimp were found to be resistant to WSSV by apoptosis-mediated blocking of viral propagation, hence hindering the virus from spreading in the host cells of shrimp [195]. However, WSSV also evolved anti-apoptotic proteins such as AAP-1 (ORF390 or WSSV449), WSV222, VP38, WSSV134, and WSSV322 that delay or inhibit normal apoptosis, allowing completion of the viral replication cycle and further infection of other cells [183].

**Figure 7:**
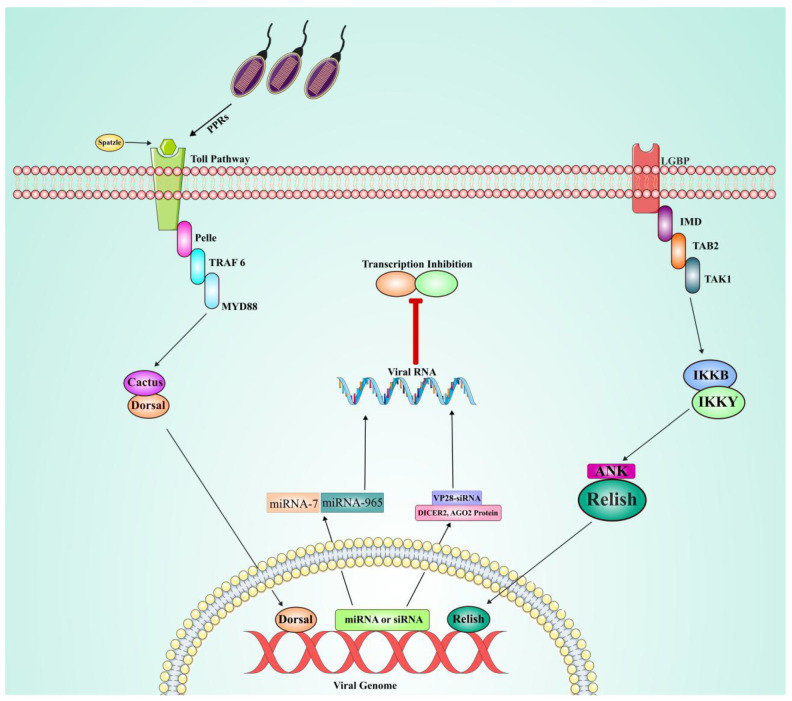
Humoral immune response mechanisms in shrimp against White Spot Syndrome Virus (WSSV) infection.

Humoral immune mechanisms in shrimp: In view of the absence of lymphocytes and immunoglobulins in shrimp, much dependence is placed on the humoral immune response as a complement to cellular immunity. Various biological macromolecules, including antimicrobial peptides, phosphatase, and lysozyme, are involved in mediating humoral immunity in shrimp against pathogen invasion [183]. It has been found that these molecules show crucial activity related to the recognition and neutralization of pathogens, thereby preventing their proliferation within the host [196]. The Toll, IMD, and JAK/STAT pathways are considered the main signaling pathways of the humoral response in shrimp, especially in their immune response to viral infections like WSSV (Figure 7). Expression of the canonical Toll pathway has been well characterized in several shrimp species, including *L. vannamei*, *P. monodon*, *M. rosenbergii*, *P. clarkii*, *F. chinensis*, and *M. japonicus* [99,197,198,199,200,201]. Key molecules in this pathway include Spätzle, Toll, MyD88, Tube, Pelle, Pellino, TRAF6, Dorsal, Cactus, Tollip, SARM, Flightless-I, and b-arrestin, each of which plays subsequent roles in the activation of immune responses [202]. Interestingly, up to date, 25 Toll-like receptor genes have been identified in shrimp, with species-specific variations. These include LvToll1-9 from *L. vannamei*, PmToll1 and PmToll9 from *P. monodon*, FcToll from *F. chinensis*, PcToll and PcToll1-5 from *P. clarkii*, MjToll1-2 from *M. japonicas*, as well as two MrTolls and MrToll1-3 from *M. rosenbergii* [99,201,203,204,205,206,207,208,209,210,211,212]. These findings highlight the evolutionary adaptation of the Toll pathway in shrimp to respond in a species-specific manner against pathogens.

The immune deficiency (IMD) pathway, first identified in *L. vannamei* in 2009 [213], also contributes to antiviral humoral immunity. Several IMD homologs known to have conserved functions have become species-specific regarding tissue distribution and immune response [214]. For example, FcIMD is mainly expressed in the stomach and gills from *F. chinensis*, while PcIMD is highly expressed in the hepatopancreas, stomach, and heart from *P. clarkia* [213]. The IMD pathway contains several canonical gene components (Relish, TAK1, TAB1, and TAB2) shared with the Toll pathways and are involved in activating immune response [184]. *L. vannamei* has been found to express LvTAK1 and LvTAK2, where these Toll-like receptors regulate the expression of various antimicrobial peptides in vivo [215]. In addition, Lvb-TrCP, LvMKK6, LvAkirin, LvNKRF, LvRelish exhibit strong responses to WSSV viral infection from *L. vannamei*. Along with PmRelish, FcRelish, FcMKK4, and FcP38 also show activity against viral infections like WSSV in the *M. japonicas*, *F. chinensis* [202].

Compared to the well-characterized Toll and IMD pathways, the role of the JAK/STAT pathway during WSSV infection remains unclear; however, recent studies have unraveled the dual function of the JAK/STAT pathway in WSSV infection. In the JAK/STAT pathway, LvSOCS2 activates the expression of antimicrobial peptides, exhibiting an antiviral role in *L. vannamei*. Conversely, LvJAK promoted infection with viral genes such as WSV069 during the early stages of WSSV infection [216]. From these observations, it seems that, in general, the JAK/STAT pathway plays a dual role (positive or negative) during a viral infection depending on the different stages of infection and specific immune factors involved.

RNA interference (RNAi) and microRNA (miRNA) in shrimp immunity: Another important aspect of shrimp immunity is that RNAi and miRNA are involved in regulating immune responses. RNAi mediated by siRNAs has been found to be an essential defense against viral infections [22]. Shrimps produce siRNAs specific to viruses, like vp28-siRNA against WSSV infection, lowering viral replication by targeting viral genes in hemocytes. The main RNAi machinery elements, Dicer2 and Argonaute2, take part in the generation and function of siRNAs in antiviral reactions [217,218]. Along with RNAi, miRNAs have also become important controllers of shrimp immune reactions [219]. Various miRNAs displaying differential expression resulting from infection by WSSV have been identified through miRNA microarray analysis. For example, miR-7 and miR-965 have been identified as down-regulators of WSSV early genes, such as wsv477 and wsv240 228], respectively, which ultimately inhibit viral replication and subsequent infection [220]. In addition, miR-965 has been shown to enhance phagocytic activity via autophagy-related gene-5, a gene involved in autophagy—thereby aiding in the phagocytosis of viral pathogens. On the contrary, some other viral miRNAs, such as WSSV-miR-66 and WSSV-miR-68, enable viral infection via the enhancement of expression levels during the early stage in WSSV infection by enhancing the expression of viral genes (wsv094, wsv177, wsv248 and wsv309) [221]. These findings illustrate the complex interplay between host and viral miRNAs in regulating immune responses during WSSV infection.

## 7. On-Going Research on Control Measures

The conventional control of WSSV using antibiotics indiscriminately failed through inefficacy and bioaccumulation problem [222], prompting the transition to new approaches such as immunostimulants, dietary interventions, nanotechnology, DNA vaccines, RNA interference, and CRISPR-Cas gene editing [223,224,225,226,227,228,229,230,231].

Immunostimulants have become a strong tool in the arsenal of enhancing immunity in shrimp against WSSV infection. Different natural substances, seaweed extracts, essential oils, probiotics, plant-based chemicals and animal-derived immunostimulants have proven their efficacy by enhancing the resistance of shrimp to viral infections through boosting innate and non-specific immunity [102,232,233,234,235,236,237,238]. One such compound is fucoidan, a sulfated polysaccharide extracted from brown algae such as *Fucus vesiculosus*. In shrimp, it enhanced immune responses significantly, with hemocyte counts, respiratory activity, and prophenoloxidase activity increasing around 40–50% compared to without treatment fucoidan [239]. All these mechanisms are capable of preventing viral replication, thereby reducing WSSV infection intensity. Another example is the sulfated galactan from red algae, which interacts with viral proteins VP26 and VP28, thereby preventing virus attachment to shrimp cells and further severely impeding its entry and replication [240]. Taken together, these findings indicate the dual activity of these compounds—immune augmentation and viral interference—and thus represent hopeful leads for non-antibiotic viral control. Polyphenolic compounds and flavonoids like epigallocatechin gallate (EGCG) and naringenin have shown up to 90% in vivo antiviral activity, varying between 40% and 90% with dosage and tissue type [241,242]. The ability of these food bioactives to modify immune gene networks holds potential for functional feed products beyond symptom management and toward the long-term viral (WSSV) immunity of shrimp. Traditional medicine plants such as *Agathi grandiflora* and *Argemone mexicana* have also displayed promising immunostimulatory activity with measurable decreases in mortality (~40%) and improved physiological indices such as reduced coagulation time (61%) and total hemocyte counts (51.82 × 10^5^ cells mL^−1^) [236,243]. Results agree with the assumption that the synergistic phytocompounds can induce broad-spectrum immune responses, although standardization of the active molecules remains a problem. Combining these findings, it is evident that immunostimulants are not merely adjuvants but rather can be fundamental components of integrated WSSV control strategies. Their ability to act at various levels such as molecular (gene expression), cellular (activation of hemocytes), and structural (viral entry prevention)—places them in a unique position to replace the gap that was left due to the unavailability of approved antiviral medications. Additional improvement in delivery systems (e.g., encapsulation) and mechanistic research will be critical to taking these bioactives from experimental-scale trials to farm-level uses. Exploring conserved viral targets like ribonucleotide reductase (RR) as antiviral lead drug candidates [76] is a complementary strategy to the immunostimulant. RR inhibition can disrupt WSSV replication at its core and, combined with immune-stimulatory molecules, can create a multi-faceted defense against viral epizootics. Combinatorial approaches such as these must receive increased experimental scrutiny and policy-level interest to stem antibiotic reliance in shrimp aquaculture.

Probiotics have gained momentum in shrimp virology because they provide a two-fold advantage of improving shrimp gut health and strengthening immune responses. Species such as *Pediococcus pentosaceus*, *Lactobacillus*, and *Bacillus* spp. have exhibited immune-stimulatory activities that are translated into protection in the field against WSSV. For example, *Lactobacillus johnsonii* KD1 reduced mortality to 38% five days post-infection—a 61% inhibition of that in untreated controls [234,244]. Probiotics like *Bacillus subtilis* and *Vibrio alginolyticus* enhance the activity of digestive enzymes and gut integrity, secondary to inhibiting WSSV replication [245,246]. The potency of probiotics is not only in immune-stimulation but potentially redressing microbial communities into less permissive environments for viral persistency—an area that demands more longitudinal studies in actual farm environments. Dietary interventions, such as the inclusion of bioactive compounds (flavonoids, oligosaccharides, carotenoids, and polyphenols) into shrimp feed, have been highly successful in conferring immunity in shrimp against WSSV. Of these, quercetin has shown specific promise—crayfish supplemented with 40 mg/kg quercetin exhibited the highest survivals following a WSSV challenge test, along with significant reductions in viral load upregulating immune-related genes (TLR, ALF, and NF-κB) [29,247,248]. Inulin at 2.5–5.0 g/kg feed enhanced phenoloxidase activity in *L. vannamei* and was effective against low-load infections [249,250,251]. Galactooligosaccharides not only altered gut microbial communities but also decreased stress markers (e.g., glucose) and enhanced phagocytic and hemocyte function, indicating broad-spectrum immune conditioning [249,250,251]. Additionally, carotenoids have been reported to elevate a variety of immune genes (TRX, PGRPs, ferritin, ProPO, TLRs, Vg), and heat shock proteins by as much as twofold [249,250,251]. Combinedly these compounds form the basis of a multilayered immune readiness state in shrimp, particularly when blended or used as functional feed preparations. Another major advantage of these dietary interventions is their efficiency in maintaining the health of the gut, thereby indirectly improving the immune system in shrimp. These dietary compounds promote nutrient digestibility and digestive enzyme secretion through maintenance of orderly gut microbiota, resulting in better growth and resilience to viral infections such as WSSV. However, the issue is standardizing dosages and delivery systems, most notably under varying aquaculture regimes. Essential oils also expand the profile of natural antivirals. Essential oils from plants such as *Zanthoxylum tsihanimposa* and *Eucalyptus globulus* were also recommended as effective natural treatments for WSSV [252,253]. Shrimp that was treated with *Zanthoxylum* extracts maintained zero mortality and zero WSSV detection at 90 days of culture, highlighting long-lasting protective effects. *Eucalyptus* oil increased levels of hemocytes from 9 × 10^6^ to 2 × 10^7^ within three days, indicating rapid immunological response. Thyme oil, when microencapsulated, also increased proPO activity of shrimp and survival against WSSV [254] and combinations of essential oils showed synergistic effects, further enhancing antiviral properties [255]. Though promising, essential oil compositions are frequently limited by issues of stability and dosing accuracy, making technological assistance such as nanoencapsulation necessary for practical use.

Apart from these nutritional and phytochemical based research, nanotechnology provides advanced solutions in the detection and prevention of WSSV. For example, the detection of WSSV in shrimp using gold nanoparticle (GNP)-based dark-field counting, which is at the same sensitivity level as qPCR (32 copies/μL) but with significantly shorter detection time (~30 min), enabling on-site detection of viral load rapidly [256]. By being very effective, cost and scalability are concerns to practical use. For prevention, PVP-coated silver nanoparticles exhibit excellent anti-WSSV activity via attachment inhibition and virucidal activities with maximum efficacy at 320 μg/mL in PmLyo-Sf9 cells [231]. Molecular docking confirms good binding affinities of VP28 envelope protein and silica nanoparticle complexes (K(AlSi_3_O_8_), Na_2_Si_2_O_5_, and SiO_2_: −5.2 to −8.3 kcal/mol) to optimize targeted viral neutralization. Promising in vitro and in silico data notwithstanding, extensive in vivo validation remains deserving. As prophylactics, polyanhydride nanoparticles are potentially very valuable, achieving ~80% protection in *L. vannamei* following a WSSV challenge test by ensuring the successful delivery of dsRNA vaccines [23]. Such sebacic acid and derivative monomers-based polymers ensure controlled release and aqueous stability. Alongside polyanhydride, nanoparticles and virus-like particles (VLPs) present another promising tool for controlling diseases in shrimp, particularly for delivering dsRNA. VLPs from Infectious Hypodermal and Haematopoietic Necrosis Virus and *Macrobrachoum rosenbergii* nodavirus capsid proteins were demonstrated to encapsulate dsRNAs (e.g., VP28, VP37) effectively, facilitating uptake, immune stimulation, and viral inhibition [229,257,258,259]. VLPs structurally resemble viruses but do not contain any viral genetic material, making them ideal for bypassing host defenses and safely transporting therapeutic dsRNA. This is because VPLs mimic the external properties of viruses and make the way to stimulate immune responses without the risk of causing infection. These findings suggest that VLPs not only protect dsRNA from breakdown within host cells but also improve the uptake and stability of these therapeutic molecules. Other systems—liposomes, chitosan, beta-glucan, and plant virus-based vectors (cowpea chlorotic mottle virus, brome mosaic virus)—are under investigation for dsRNA delivery, with varying biophysical and immunological properties [260,261,262,263,264]. Nanotechnology is a promising alternative to conventional control methods; however, comparative studies, regulatory clarity, and environmental risk analyses will still be required to provide safe inclusion in shrimp production systems.

One of the most promising recent developments in WSSV control involves the use of DNA and RNA vaccines. DNA vaccines targeting conserve viral proteins such as VP28 have been reported to enhance immune enzyme activity (super-oxide dismutase (SOD) and alkaline phosphatase (AKP)) significantly and enhance shrimp survival [257,265,266]. These vaccines, especially when formulated based on conserved genomic regions, are of high translational value because they are likely to be efficacious in the long term despite genetic heterogeneity of WSSV (discussed earlier in Section 4.2). This method is very strong, especially with climate-driven viral evolution emphasizing the requirement for stable antigen targets. Chitosan coating on DNA vaccines further improves oral delivery and stability, making them convenient to use in the field [267,268], although further comparative trials must be carried out to establish that they have a benefit over traditional injection methods. On the contrary, RNA vaccines facilitate gene-level targeting but suffer from a major drawback: instability of double-stranded RNA in aquatic systems [23]. This has steered attention towards RNA interference (RNAi), wherein dsRNAs against some key viral genes of WSSV (VP19, VP28, rr1 and rr2) [257,258,269,270] or even host elements like PmRab7, PmRab7/PmIAP and GFP have shown enormous viral suppression—95% in the case of PmRab7 + rr2 [271,272]. These researchers exhibit great potential with RNAi-based technologies. However, one of the main drawbacks lies in delivery effectiveness and stability within real aquaculture conditions. In my opinion, linking these molecules with guardian nanocarriers, as explained earlier, is a viable solution. However, until these RNA-based products demonstrate consistent results through field testing and receive approval from regulatory agencies, their widespread adoption will significantly continue to be restricted.

CRISPR-Cas gene editing has also opened up new avenues to enhance the resistance of shrimp to WSSV by precision genomic interventions. It entails editing shrimp immune-suppressive genes such as GIH and MIH, which, upon downregulation, can promote host immunity [228,273,274,275]. While the theoretical background is fascinating, practical application continues to be plagued by technical as well as ethical constraints, especially for germline edits in aquaculture organisms. One intriguing point is the possibility of CRISPR systems delivering viral DNA as “spacers” into the shrimp genome to confer adaptive antiviral immunity akin to bacterial immunity—a topic worthy of exploration, albeit presently hypothetical in crustaceans [228]. Along with this, transcriptomic research identified key immune-related genes and pathways induced with WSSV infection, with implications for the possibility of dietary or functional feed interventions to modulate gene expression for enhanced resistance. At another level, proteomic research has identified the differential expression of antiviral proteins such as proPO, lysozyme, and crustin in WSSV infection [276,277]. While such reports are helpful, WSSV research based on omics is still in its infancy, and there is a wide gap in functional characterization of structural proteins for vaccine development. Integrating CRISPR to omics data can revolutionize targeted breeding or immune-priming strategies; however, field-ready technologies will require technological development as well as stronger regulatory frameworks. In the interim, such technologies are intellectually appealing but practically in their infancy.

The growing threat of WSSV in shrimp farming has necessitated the development of alternative control strategies beyond traditional methods like antibiotics, which have proven ineffective and harmful. Recent advances in immunostimulants, dietary interventions, probiotics, essential oils, DNA vaccines, nanotechnology, and CRISPR-Cas gene editing offer promising solutions for enhancing shrimp immunity and preventing WSSV outbreaks. The continued integration of these technologies, along with a deeper understanding of shrimp immune responses through transcriptome and proteomic analyses, holds the key to future breakthroughs in combating WSSV.

## 8. Future Research Direction

WSSV is one such pathogen, and the battle against it in shrimp aquaculture underscores the urgency for novel means of control that are environmentally friendly and effective. Although WSSV was one of the first shrimp viruses to be studied in depth, it remains one of the major viral agents affecting shrimp farming worldwide, causing severe economic losses and food security concerns. In this review, we have illustrated the current knowledge of WSSV, with a special focus on virus sensing and manipulation, spread mechanisms, and the strategies being applied in aquaculture to ensure biosecurity, induce immunity, and apply biotechnology against WSSV. Yet, much more needs to be done to address unanswered questions and to develop scalable, long-lasting solutions.

Genome-editing approaches such as CRISPR/Cas9, specifically for WSSV resistance could facilitate the development of new shrimp lines with improved immunity. There is the need for more functional studies to identify not only resistance-related genes but also epigenetic traits that control resilience at a phenotypic level—traits that may confound outcomes related to WSSV susceptibility. Furthermore, exploring the interactions between environmental stressors such as water quality and temperature with genetic resistance could facilitate holistic management approaches that enhance disease resilience.

Shrimp immunology remains under-explored in the context of WSSV control. Since shrimp lack an adaptive immune system like vertebrates, innate immunity is essential. In conclusion, future studies on the molecular mechanism of immune stimulants and probiotics should focus on developing formulations that induce optimal and safe outcomes against WSSV. Despite advances in improving shrimp immunity, extensive research is still needed to determine the optimal doses, timing, and combinations of plant extracts and marine probiotics as immunostimulants. Moreover, these natural immunostimulants alone could be coupled with newly available genetic tools to create novel and complementary strategies for strengthening shrimp populations against WSSV.

WSSV control through biotechnology still holds promise. Though DNA and RNA-type vaccines show great potential, the socio-economic hurdles remain for their large-scale commercial use. Newer generation vaccines, including nanoparticle and oral formulations, may overcome some of these constraints and make vaccination feasible in open aquaculture systems. Research should also explore delivery methods that are stable enough for use in the field, perhaps through embedding RNAi agents within feeds or devising a time-release nanoparticle system that can be disseminated into shrimp ponds. Additionally, studying WSSV genome evolution under the selective pressure of such control measures could help predict and prevent the development of viral resistance.

The introduction of nanotechnology, although still in its early stages for WSSV control, offers a range of possibilities for both detection and treatment. Detection tools that use nanoparticle sensors could provide rapid diagnostics in shrimp ponds, allowing farmers to respond before outbreaks develop. Moreover, nanoscale technology may also facilitate the delivery of antiviral compounds or RNAi-based therapeutics, increasing their stability and bioavailability. Nevertheless, studies are needed to assess the safety and environmental sustainability of nanomaterials in aquaculture systems, where biosecurity measures often impact the efficacy/success of transmission-related tools used for the mitigation of shrimp viruses at a large scale.

Another challenge in WSSV control is the limited understanding of its transmission pathways, including the role of wild crustacean carriers and environmental reservoirs. Vertical transmission through waterborne exposure is known to be highly effective, but little is known about the environmental persistence of WSSV under varying conditions such as salinity, pH, and temperature status. Information from field studies should be further exploited to assess how these variables affect viral persistence, enabling the design of biosecurity measures tailored to local climatic and ecological circumstances. Research into other polymicrobial environments involving WSSV and other pathogens may also uncover synergistic interactions that affect the severity and spread of the infection. This knowledge would also be useful for creating compartmentalized aquaculture systems that could limit disease spread without the use of antibiotics.

We believe that the path towards well-informed WSSV management in shrimp aquaculture will be shaped by interdisciplinary efforts and ongoing challenges. Integration across genetic resistance, immunological studies, biotechnology, and ecological research can result in multifaceted, durable solutions to WSD. To ensure that these innovations translate into real-world impact, researchers, industry stakeholders, and policymakers must work together to bridge the gap between laboratory findings and field applications. As shrimp aquaculture continues to expand globally, it must balance environmental sustainability with robust viral biosecurity practices. These two forces must operate in tandem, not in isolation, to maintain long-term stability and productivity.

## Figures and Tables

**Table 1:** Recent outbreak of WSSV in different countries around the world [50,51].

Country/Region	Year	Country	Year
Mozambique	2019	Ecuador	2019
China	2019	Bangladesh	2012
India	2018	Brunei Darussalam	2013
Indonesia	2019	Colombia	2005
Japan	2020	El Salvador	2005
Malaysia	2009	Honduras	2013
The Philippines	2019	Hong Kong	2013
South Korea	2019	Iran	2013
Taiwan	2019	Madagascar	2013
Thailand	2019	Myanmar (Burma)	2012
Vietnam	2019	Nicaragua	2013
Costa Rica	2019	Peru	2013
Mexico	2019	Iran	2011
Panama	2019	Saudi Arabia	2012
United States	2020	Venezuela	2011
Australia	2020	Argentina	2010
Brazil	2019		

**Table 2:** Host species reported to be naturally or experimentally infected with WSSV [83,105,106].

Scientific Name	Common Name	Type	Detection Method
***Alpheus brevicristatus***	Snapping shrimp	N	Nested PCR
*Alpheus lobidens*	Apping shrimp	N	Nested PCR
*Aristeus* sp.	Red shrimp	N	Nested PCR
*Exopalaemon orientalis*	Oriental prawn	N, E	Nested PCR, DNA Probe
*Penaeus aztecus*	Northern brown shrimp	N, E	PCR, Histo
*Penaeus duorarum*	Pink shrimp	N, E	Histo
*penaeus penicillatus*	Rod Tail Shrimp	N	Nested PCR
*penaeus chinensis*	Chinese white shrimp	N	Histo, Electron microscope
*Penaeus vannamei*	Whiteleg shrimp	N, E	TEM, PCR, Histo
*Penaeus setiferus*	Atlantic white shrimp	N, E	PCR, Histo
*Macrobrachium rosenbergii*	Giant freshwater shrimp	N, E	Nested PCR, Southern blot, Histo
*Macrobrachium idella*	Sunset shrimp	E	Southern blot, Histo
*Marsupenaeus japonicus*	Kuruma shrimp	N, E	TEM, PCR, Histo
*Metapenaeus ensis*	Greasyback shrimp	N, E	PCR, DNA Probe
*Metapenaeus dobsoni*	Kadal shrimp	N, E	PCR, DNA Probe
*Metapenaeus lysianassa*	Bird shrimp	N	PCR, Histo
*Metapenaeus monoceros*	Speckled shrimp	N, E	Nested PCR, DNA Probe, Histo
*Metapenaeus elegans*	Fine shrimp	N	Nested PCR
*Palaemon adspersus*	Baltic prawn	E	TEM, Dot Blots, ISH, 1-step PCR
*Palaemon styliferus*	Grass shrimp	N	Nested PCR, DNA Probe, Histo
*Parapenaeopsis stylifera*	Kiddi shrimp	N	Nested PCR, DNA Probe
*Penaeus monodon*	Giant tiger shrimp	N, E	TEM, PCR, Histo
*Penaeus indicus*	Indian white prawn	N, E	TEM, Histo
*Penaeus merguiensis*	Banana prawn	N	ISH, Histo
*Penaeus semiculcatus*	Green tiger prawn	N, E	PCR
*Penaeus schmitti*	Southern white shrimp	E	Histo, ISH
*Penaeus duorarum*	Northern pink shrimp	N/E	Histo
*Penaeus stylirostris*	Northern white shrimp	N/E	TEM, PCR, Histo
*Solenocera indica*	Coastal mud shrimp	N	PCR, DNA Probe
*Trachypenaeus curvirostris*	Southern rough shrimp	N, E	LAMP, 2-step PCR
*Atergatis integerrimus*	Bashful crab	E	PCR, Histo
*Cancer pagurus*	Edible or rock crab	E	TEM, ISH, Histo, 1-step PCR
*Calappa lophos*	Box crab	N, E	PCR
*Calappa philargius*	Box crab	E	PCR, Histo
*Callinectes arcuatus*	Swimming crab	N	PCR
*Callinectes sapidus*	Blue crab	N	ISH, PCR
*Carcinus maenas*	Littoral crab	E	TEM, DNA hybridization, PCR
*Charybdis annulata*	Swimming crab	N, E	Histo, PCR
*Charybdis cruciata*	Red sea crab	N	PCR
*Charybdis feriata*	Coral crab	E	Nested PCR
*Charybdis granulata*	Swimming crab	E	Nested PCR
*Charybdis hoplites*	Swimming crab	N	PCR
*Charybdis lucifera*	Swimming crab	N, E	Histo, PCR
*Charybdis natator*	Hairyback crab	N	Histo, PCR
*Demania splendida*		E	Histo, PCR
*Doclea hybrida*		E	Histo, PCR
*Gelasimus marionis nitidus*		N	PCR
*Grapsus albolineatus*	Rock crab	E	Histo, PCR
*Halimede ochtodes*	Hairy crab	E	Histo, PCR
*Helice tridens*	Shore crab	N	2-step PCR
*Liocarcinus depurator*	Harbor crab	E	TEM, Dot blots, ISH, PCR
*Liocarcinus puber*	Velvet swimming crab	E	TEM, Dot blots, ISH, PCR
*Lithodes maja*	Deepsea king crab	E	Histo, PCR
*Macrophthalmus sulcatus*	Ghost/fiddler crab	N	PCR, DNA Probe
*Mantura* sp.		N	PCR
*Matuta miersi*	Moon crab	E	Histo, PCR
*Matuta planipes*	Moon crab	N	PCR
*Metopograpsus messor*	Purple climber crab	N	PCR, DNA Probe
*Menippe rumphii*	Stone crab	E	Histo, PCR
*Paradorippe granulata*		E	Histo, PCR
*Parthenope prensor*	Elbow crab	E	Histo, PCR
*Parathelphusa hydrodomous*		E	PCR, Histo
*Parathelphusa pulvinata*		E	PCR, Histo
*Philyra syndactyla*	Purse crab	E	PCR, Histo
*Podophthalmus vigil*	Long-eyed swimming crab	E	PCR, Histo
*Portunus pelagicus*	Sand crab	N, E	PCR
*Portunus sanguinolentus*	Blood spot crab	N, E	PCR, Histo
*Pseudograpsus intermedius*	Mosaic crab	N	Nested PCR, DNA Probe, Histo
*Scylla serrata*	Mud crab	N, E	PCR, Histo
*Scylla tranquebarica*	Mangrove crab	N, E	PCR, TEM
*Scylla olivacea*	Orange mud crab	E	qPCR
*Sesarma* sp.	Marsh crabs	N, E	PCR, Histo
*Somanniathelphusa* sp.	Black rice crab	E	PCR, Histo
*Thalamita danae*	Swimming crab	E	PCR, Histo
*Acetes* sp.	Krill	E	PCR, Histo
*Panulirus homarus*	Scalloped spiny lobster	E	Histo, Bioassay
*Panulirus longipes*	Longlegged spiny lobster	E	Nested PCR
*Panulirus ornatus*	Ornata spiny lobster	E	Histo, Bioassay
*Panulirus penicillatus*	Pronghorn spiny lobster	N, E	PCR
*Panulirus polyphagus*	Mud spiny lobster	E	Histo, Bioassay
*Panulirus versicolor*	Painted spiny lobster	E	Nested PCR
*Scyllarus arctus*	Small European locust lobster	E	TEM, Dot Blots, ISH, PCR
*Artemia*		E	Nested PCR
*Artemia franciscana*		E	Nested PCR
*Schmackeria dubia*	Copepoda	N	PCR
*Squilla mantis*	Mantis shrimp	N	Nested PCR, DNA Probe
*Marphysa gravelyi*	Polychaeta	N	2-step PCR
*Brachionus urceus*	Rotifera	E	Nested PCR
*Ephydridae* sp.	Shore fly	N	Nested PCR
*Astacus leptodactylus*	Turkish crayfish	E	TEM, Dot Blots, ISH, 1-step PCR
*Astacus astacus*	Broad-fingered crayfish	E	PCR
*Cherax destructor albidus*	Yabby	E	DNA Probe, Histo
*Cherax quadricarinatus*	Australian redclaw	E	TEM, ISH, Nested PCR
*Orconectes limosus*	Spinycheek crayfish	E	TEM, Dot Blots, ISH, 1-step PCR
*Orconectes punctimanus*	Spothanded Crayfish	N	DNA Probe, southern blot, PCR
*Pacifastacus leniusculus*	Signal crayfish	E	Histo, PCR, ISH
*Procambarus clarkii*	Red swamp crayfish	E	Histo, PCR

ISH—in situ hybridization, Histo—histopathology, TEM—transmission electron microscope, N—natural infection, E—experimental infection, LAMP—loop-mediated isothermal amplification.

**Table 3:** Identified WSSV proteins and their gene origins.

Protein Names	A. A ** Residues Size	Apparent Size (kDa)	Location in WSSV Virion	WSSV-CN ORF	References
VP187	1606	174	Envelope	wsv209	[109]
VP180	1684	169	Envelope	wsv001	
VP150	1301	144	Envelope	wsv011	
VP136B	1243	136	Envelope	wsv465	
VP124	1219	136	Envelope	wsv216	
VP110	972	110	Envelope	wsv035	
VP90	856	96	Envelope	wsv327	
VP75	786	75	Envelope	wsv332	
VP56 (VP60A)	465	60	Envelope	wsv325	
VP55	448	55	Envelope	wsv526	
VP53A	1301	144	Envelope	wsv011	
VP53B	968	53	Envelope	wsv115	
VP52A	486	51	Envelope	wsv238	
VP38	283	32	Envelope	wsv259	
VP33 (VP36B)	281	32	Envelope	wsv254	
VP32	278	32	Envelope	wsv198	
VP28	204	28	Envelope	wsv421	
VP22	891	100	Envelope	wsv303	
VP19	121	19	Envelope	wsv414	
VP13A	100	13	Envelope	wsv284	
VP12 (VP12A)	95	11	Envelope	wsv009	
VP12B	68	7	Envelope	wsv386	
VP11	433	11	Envelope	wsv338	
WSSV189	-	-	ND *	wsv134	
WSSV471	-	-	ND *	wsv412	
VP95	95	11	Tegument	wsv442	
VP39A	419	39	Tegument	wsv306	
VP36A	297	36	Tegument	wsv077	
VP26	204	26	Tegument	wsv311	
VP24	208	24	Tegument	wsv002	
WSSV458	-	-	ND *	wsv399	
WSSV186	-	-	ND *	wsv131	
VP160B	1280	143.8	Nucleocapsid	wsv037	[113]
VP53C	489	53	Envelope	wsv269	[111]
VP52B (VP51B)	384	46	Envelope	wsv256	
VP41A	292	33	Envelope	wsv237	
VP41B	300	34	Envelope	wsv242	
VP39 (VP39B)	419	39	Envelope	wsv339	
VP38B	309	35	Envelope	wsv390	
VP31	261	31	Envelope	wsv340	
VP13 (VP13B)	117	13	Envelope	wsv321	
VP14	97	11	Envelope	wsv293a	
VP664	6077	664	Nucleocapsid	wsv360	
VP190	1565	174	Nucleocapsid	wsv289	
VP136	1218	135	Nucleocapsid	wsv271	
VP60 (VP60B)	544	62	Nucleocapsid	wsv415	
VP51 (VP51C)	466	62	Nucleocapsid	wsv308	
VP15	80	15	Nucleocapsid	wsv214	
VP76 (VP73)	674	76	Nucleocapsid	wsv220	[114]

* ND—Not Determined, ** A. A—Amino acids.

**Table 4:** Gross clinical signs of WSSV in various species.

Species	Clinical Sign Observed	Reference
*Penaeus monodon*	White spots, lethargy, soft shell, erratic swimming, reddish body	[11]
*Penaeus vannamei*	White patches, gill necrosis, soft cuticle, lethargy	
*Marsupenaeus japonicus*	White spots, disorientation, loose appendages, lethargy	
*Penaeus chinensis*	Soft body, white spots, gill necrosis, lethargy	[101]
*Scylla olivacea*	White spots on carapace, necrosis, lethargy	[141]
*Neohelice granulata*	White spots, lethargy, body discoloration	
*Procambarus clarkii*	White spots, abnormal swimming, lethargy	[142]
*Macrobrachium rosenbergii*	Red body, gill necrosis, lethargy	[138]
*Metapenaeus ensis*	Lethargy, body redness, white spots	[83]
*Exopalaemon orientalis*	White spots, soft exoskeleton, decreased mobility	
*Calappa lophos*	Lethargy, body discoloration, white spots	

**Table 5:** Histopathological degeneration of different organs in WSSV-affected shrimp.

Target Organ	Histopathological Degeneration	Reference
Epidermis and cuticular epithelium	Nuclear hypertrophy, intranuclear inclusion bodies, necrosis, epithelial detachment	[149]
Gills	Lamellar sloughing, epithelial fusion, necrosis, presence of viral inclusion bodies	[150]
Lymphoid organ and haematopoietic tissues	Formation of LOS, haemocyte infiltration, apoptotic bodies	[151]
Hepatopancreas and digestive system	Hepatopancreatic tubular degeneration, bacterial co-infection, severe necrosis in midgut epithelium	[152]
Y-organ (endocrine gland)	Destruction of molting gland cells, hypertrophic changes affecting growth and reproduction	[153]

**Table 6:** Interactions between homologous and heterologous pathogens that co-infect different shrimp species.

Host Species	First Pathogen	Second Pathogen	Type of Infection	Reference
Homologous co-infection				
	Viral co-infections			
*Litopenaeus vannamei*	Infectious hypodermal and haematopoietic necrosis virus (IHHNV)	White Spot Syndrome Virus (WSSV)	Synergistic	[150,165]
*Penaeus monodon*	IHHNV	WSSV	Synergistic	[166]
*Penaeus monodon*	WSSV	Monodon baculovirus	Antagonistic	[167,168]
*Penaeus monodon*	Monodon baculovirus	IHHNV		
*Penaeus monodon*	Monodon baculovirus	Hepatopancreatic parvovirus	Synergistic	[169]
*Penaeus monodon*	WSSV	*Penaeus stylirostris* densovirus	Synergistic	[170]
*Litopenaeus vannamei*	Infectious myonecrosis virus	WSSV	Synergistic	[171]
*Penaeus monodon*	Hepatopancreatic parvovirus	WSSV	Antagonistic	[172]
*Penaeus monodon*	Hepatopancreatic parvovirus	IHHNV	Antagonistic	
	Bacterial co-infections			
*Litopenaeus vannamei*	*Vibrio parahaemolyticus*	*Vibrio harveyi*	Synergistic	[173]
	Parasitic co-infections			
*Penaeus monodon*	Microsporidian	Gregarine	Synergistic	[174]
**Heterologous co-infection**				
	Parasitic and bacterial co-infections			
*Macrobrachium rosenbergii*	*Metschnikowia bicuspidata*	*Enterococcus faecium*	Synergistic	[175]
*Penaeus vannamei*	*Enterocytozoon hepatopenaei*	*Vibrio parahaemolyticus*	Synergistic	[176,177]
*Penaeus vannamei*	*Enterocytozoon hepatopenaei*	*V. campbellii*	Antagonistic	
	Parasitic and viral co-infections			
*Penaeus vannamei*	*Enterocytozoon hepatopenaei*	WSSV	Synergistic	[178]
*Penaeus vannamei*	Infectious myonecrosis virus	*Enterocytozoon hepatopenaei*	Synergistic	[29]
*Penaeus vannamei*	*Enterocytozoon hepatopenaei*	Taura syndrome virus	Synergistic	[179]
*Penaeus vannamei*	*Enterocytozoon hepatopenaei*	Hepatopancreatic parvovirus	Synergistic	[180]
*Penaeus monodon*	Monodon baculovirus	Microsporidian	Synergistic	[174]
	Monodon baculovirus	Gregarine		
	Hepatopancreatic parvovirus	Microsporidian		
	Bacterial and viral co-infections			
*Penaeus monodon*	*Vibrio parahaemolyticus*	WSSV	Synergistic	[181]

## Data Availability

The original contributions presented in this study are included in the article. Further inquiries can be directed to the corresponding author.
